# Biochemical Analysis of CagE: A VirB4 Homologue of *Helicobacter pylori* Cag-T4SS

**DOI:** 10.1371/journal.pone.0142606

**Published:** 2015-11-13

**Authors:** Mohd Shariq, Navin Kumar, Rajesh Kumari, Amarjeet Kumar, Naidu Subbarao, Gauranga Mukhopadhyay

**Affiliations:** 1 Special Centre for Molecular Medicine, Jawaharlal Nehru University, New Delhi, India; 2 School of Life Sciences, Jawaharlal Nehru University, New Delhi, India; 3 Department of Biotechnology, All India Institute of Medical Sciences, New Delhi, India; 4 School of Computational and Integrative Sciences, Jawaharlal Nehru University, New Delhi, India; Institut Pasteur Paris, FRANCE

## Abstract

*Helicobacter pylori* are among the most successful human pathogens that harbour a distinct genomic segment called *cag* Pathogenicity Island (*cag*-PAI). This genomic segment codes for a type IV secretion system (Cag-T4SS) related to the prototypical VirB/D4 system of *Agrobacterium tumefaciens (Ag)*, a plant pathogen. Some of the components of Cag-T4SS share homology to that of VirB proteins including putative energy providing CagE (HP0544), the largest VirB4 homologue. In *Ag*, VirB4 is required for the assembly of the system, substrate translocation and pilus formation, however, very little is known about CagE. Here we have characterised the protein biochemically, genetically, and microscopically and report that CagE is an inner membrane associated active NTPase and has multiple interacting partners including the inner membrane proteins CagV and Cagβ. Through CagV it is connected to the outer membrane sub-complex proteins. Stability of CagE is not dependent on several of the *cag*-PAI proteins tested. However, localisation and stability of the pilus associated CagI, CagL and surface associated CagH are affected in its absence. Stability of the inner membrane associated energetic component Cagβ, a VirD4 homologue seems to be partially affected in its absence. Additionally, CagA failed to cross the membrane barriers in its absence and no IL-8 induction is observed under infection condition. These results thus suggest the importance of CagE in Cag-T4SS functions. In future it may help in deciphering the mechanism of substrate translocation by the system.

## Introduction

Cytotoxin associated genes Pathogenicity Island (*cag*-PAI), is a hallmark of the Type I strains of *Helicobacter pylori (Hp)*. These strains are virulent and associated with most of the gastro-duodenal diseases, including chronic gastritis, peptic ulcer, MALT lymphoma and gastric adenocarcinoma [1–3]. CagA, the key effector molecule is the main signature of the *cag*-PAI positive strains. The complete *cag*-PAI is ~37 kb long, and encodes 27 genes including CagA which is transported into the host epithelial cells through a type IV secretion system called Cag-T4SS. In comparison to the other T4SS, Cag-T4SS shows limited sequence similarity to other T4SS [4]. Significant sequence similarities, however, are found in CagE to VirB4, CagX to VirB9, CagY to VirB10, Cagα to VirB11 and Cagβ to VirD4 [4]. Additionally, some of the Cag components are reported to be homologues of the prototypical T4SS of *Agrobacterium tumefaciens* (*Ag*) based on topology, localisation and functions. These include Cagγ (VirB1), CagC (VirB2), CagL (VirB5), CagW (VirB6), CagT (VirB7) and CagV (VirB8) [4–8]. Rest of the Cag components like Cagδ, CagZ, CagU, CagM, CagN, CagI, CagH, CagG, CagF and CagD are unique in nature [9].

T4SS are ancestrally related to the bacterial conjugation machinery and are the most versatile in transporting macromolecules across the membranes in Gram-positive and Gram-negative bacteria [10]. The prototypical T4SS of *Ag* consists of a large macromolecular assembly formed by 11 different VirB proteins (VirB1 to VirB11) and the coupling protein VirD4 [10]. The system encodes three ATPases VirB4, VirB11 and VirD4, which provide energy for pilus assembly and DNA/protein transport in addition to the core structural components [11–15]. The VirB4 proteins are highly conserved in the T4SS and are essential for substrate translocation and virulence [16,17]. The VirB4 family of proteins consists of two distinct domains: a large, well-conserved C terminal domain (CTD) that contains Walker A and Walker B motifs, and a less conserved N terminal domain (NTD). Depending on the species, the NTD contains predicted trans-membrane helix [14,18]. Recently the crystal structure of a VirB4 homologue from *Thermoanaerobacter pseudoethanolicus* has been reported [19]. Previously based on crystal structure of TrwB, the coupling protein of conjugative plasmid pR388 from *E*. *coli*, a molecular model of CTD of *Ag* VirB4 has been proposed [20]. This model predicted conservation of functionally and structurally important residues between VirB4 and TrwB [20].

Although VirB4 ATPases have characteristic Walker A and Walker B motifs, until recently no ATPase activity has been experimentally demonstrated for any VirB4 homologues of protein translocating T4SS [21]. However, two recent studies have reported ATPase activities of VirB4 homologues TrwK and TraB of conjugative plasmids pR388 and pKM101 from *E*. *coli* [14,15]. The ATPase activities of the tested VirB4 proteins depend on the solution conditions and the oligomerisation state of the proteins [14,15].

Several interactions have been reported for VirB4 of *Ag*. It interacts directly with VirB8 in the inner membrane, and its presence is necessary for the stabilisation of VirB8 [22]. VirB4 also interacts with the transglycosylase VirB1 [23]. VirD4 ATPase (coupling protein) and the bitopic membrane protein VirB10 are also known to interact with VirB4 in the prototypical T4SS [24].

However, practically nothing is known about the VirB4 homologue of Cag-T4SS CagE, except its C-terminal sequence analysis [21]. To determine the functions of CagE, in the present study, we have characterised the protein. We present the first experimental evidence of ATPase activity of any known VirB4 homologue of protein transporting T4SS. We also demonstrate the key role that CagE plays in Cag-T4SS pilus biogenesis, especially in the localisation and stabilisation of the pilus-associated components CagI, CagL and the surface protein CagH. Contribution of the protein in substrate translocation through the secretion system and IL-8 induction is also being demonstrated.

## Results

### Cellular localisation of CagE

**Fig 1:**
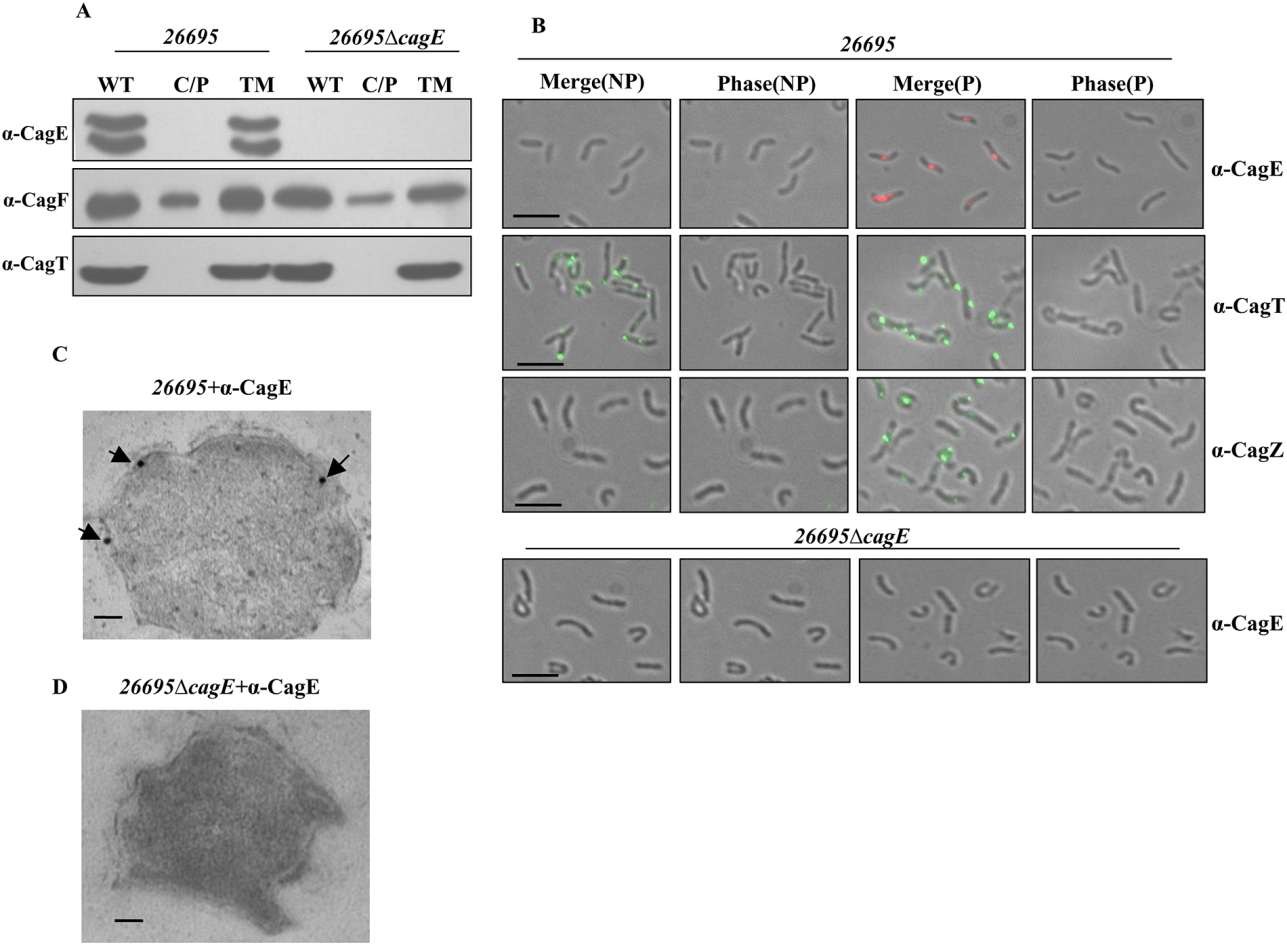
Sub-cellular localisation of CagE. **(A)** Western blots showing sub-cellular localisation of CagE in wild-type *Hp (26695)* and *26695*Δ*cagE* cells. WT, C/P, and TM indicate whole cell lysate, soluble (cytoplasmic/periplasmic) and total membrane fractions respectively. Western blots were probed with anti-CagE, anti-CagF and anti-CagT antibodies as indicated. **(B)** IFM showing CagE in wild-type *Hp (26695)* and *26695*Δ*cagE* cells under permeabilised (P) and non-permeabilised (NP) conditions. Cells were probed with the anti-CagE, anti-CagT and anti-CagZ antibodies as indicated. CagT was used as a control for surface exposed proteins and CagZ was used as a control for inner membrane proteins. *26695ΔcagE* cells were used as a negative control for anti-CagE antibody. Alexa fluor 594 (red colour) and Alexa fluor 488 (green colour) conjugated secondary antibodies were used to visualise the signals. [Out of 500 cells having fluorescent foci tested 100 foci were detected at the poles, 220 were at the middle and remaining 180 foci were detected near the poles]. Scale bars indicate 5 μm. **(C)** TEM showing inner membrane association of CagE in wild-type *Hp (26695)*. **(D)** *26695ΔcagE* cells stained with anti-CagE antibody. Cells were grown on BHI agar plates and immunogold labelling of ultrathin sections were performed as described in the materials and methods. Wild-type *Hp 26695* and *26695*Δ*cagE* (negative control) cell sections were probed with anti-CagE antibody and gold-labelled secondary antibody. Scale bars indicate 100 nm. Arrows indicate the location of gold-labelled CagE.

The prototypical VirB4 of *Ag* is an inner membrane associated NTPase. Since CagE (HP0544) being the largest VirB4 homologue, we first examined its cellular localisation by cell fractionation, immunofluorescence microscopy (IFM) and transmission electron microscopy (TEM). Wild-type (WT) *Hp 26695* and isogenic *26695*Δ*cagE* mutant (negative control) cell extracts were fractionated by ultracentrifugation as described in the materials and methods section into two major fractions: the soluble fraction (C/P), containing cytoplasmic/periplasmic contents, and the total membrane (TM) fraction. Equal volume of each fraction was separated in SDS-PAGE and Western blotted using anti-CagE, anti-CagF and anti-CagT antibodies. Western blot analysis of wild-type cells showed that CagE and CagT were exclusively present in the total membrane fraction (TM), while CagF was found in both the soluble and TM fractions (Fig 1A). Unexpectedly, however, two CagE specific bands were observed. These protein bands could not be non-specific since they disappear in isogenic *26695*Δ*cagE* mutant strain tested (Fig 1A). Same pattern of CagE was also observed in wild-type *Hp P12* and *P12ΔcagE/cagE* complemented strains (see in later sections). In this connection Kutter et al., reported that CagE is a fusion protein of VirB3 and VirB4 [8]. They showed that the first 150 amino acid of CagE has a weak similarity with VirB3 but the motifs are conserved [8]. Similarly, Mossey et al., showed a CagE type protein pattern in Western blot analysis when prototypical *virB3* and *virB4* genes of *Ag* are fused together and expressed [25]. Similarly, VirB3-VirB4 fusion proteins are reported in number of other bacteria including *Campylobacter* [26–28]. Based on the available literature and our data, we propose that the slower migrating upper band is the VirB3-VirB4 fusion protein while the faster migrating lower band is processed VirB4 alone (Fig 1A). CagT and CagF were used as membrane and soluble protein markers respectively [8,29,30].

Next, to corroborate the above result, immunofluorescence microscopic (IFM) analysis of permeabilised (P) and non-permeabilised (NP) *Hp* cells was performed as described in the materials and methods. As shown in Fig 1B, CagE was observed mostly as punctuated foci in the permeabilised cells, and no fluorescence signal of CagE was detected under non-permeabilised condition. CagT was used as a surface-exposed marker protein, while CagZ was used as an inner membrane control protein [31,32]. Taken together, these findings suggest that CagE is localised inside the bacterium attached to the membrane. Further, to visualise its exact location we performed transmission electron microscopy (TEM) and observed that it is associated with the bacterial inner membrane (Fig 1C). We used *26695*Δ*cagE* as a negative control, as expected no CagE specific signal was detected (Fig 1D). These results strongly suggest that CagE resides inside the bacterium and is associated with the inner membrane.

### Stability of CagE

**Fig 2:**
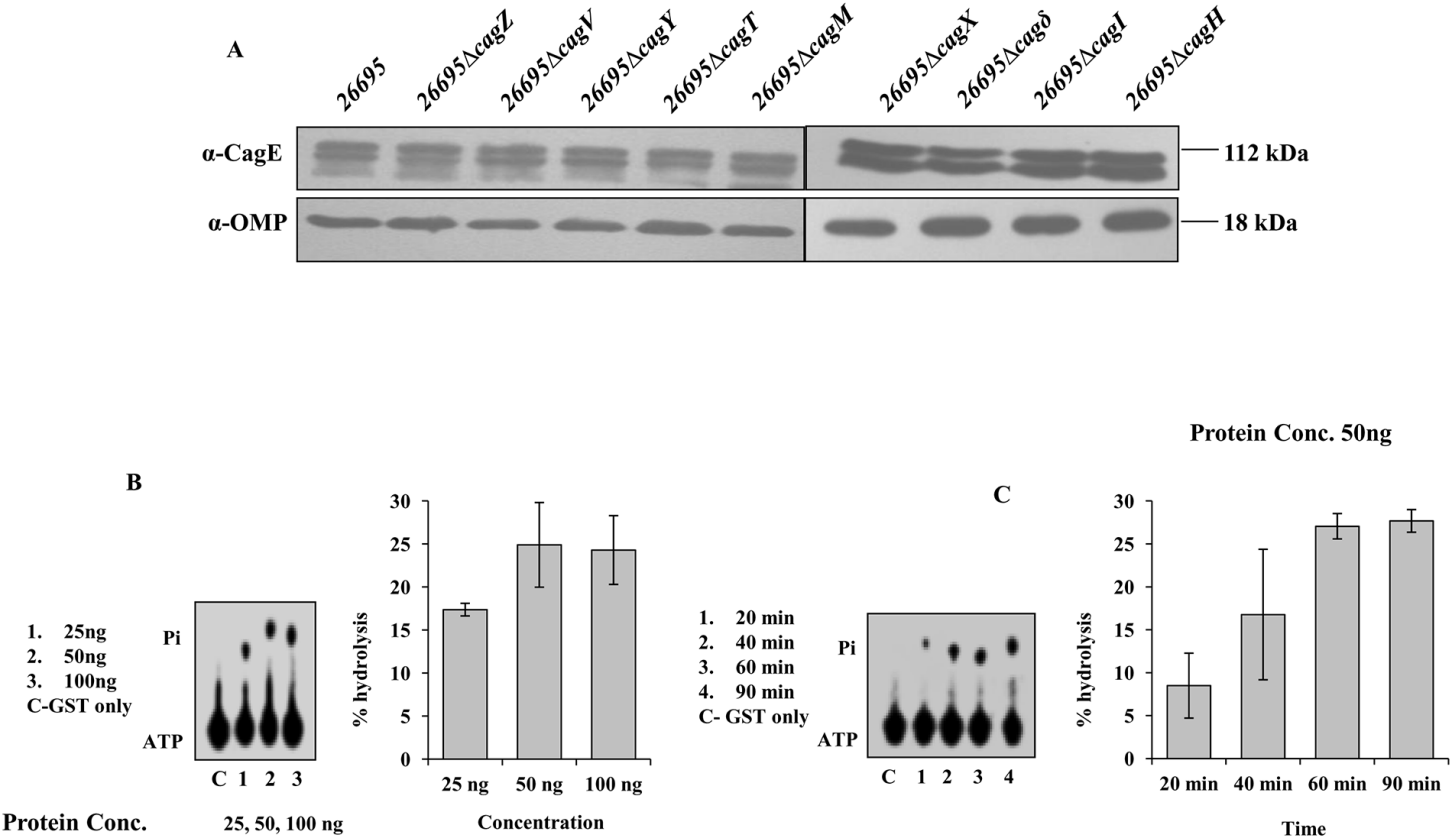
Western blots showing stability of CagE in several isogenic Cag mutants of *Hp 26695* and ATPase activity of the CTD of CagE. **(A)** Western blots showing stability of CagE in indicated strains. Antibodies used in Western blotting are indicated. OMP was used as loading control. **(B)** Concentration-dependent ATPase activity of the purified GST-CagEC (541–983, aa). Lane C: reaction with GST only; lanes 1–3: reactions with increasing concentrations of the protein as indicated. Positions of ATP and released Pi are indicated. **(C)** Time-dependent ATPase activity of the purified GST-CagEC (541–983, aa). Lane C: reaction with GST only; lanes 1–4: reactions with increasing time as indicated. Positions of ATP and Pi are indicated. Percent hydrolysis of ATP was plotted against protein concentration or against incubation time. Statistical error bars are indicated.

Several studies have shown that the stability of one or more components in multi-protein complexes depends on the presence of other components, and this phenomenon is well documented in the prototypical T4SS of *Ag* [33]. Likewise, in *Hp* Cag-T4SS, the level of CagT, a VirB7 homologue was observed to be significantly reduced in isogenic *Hp*Δ*cagX* and *Hp*Δ*cagM* mutants compared to the wild-type *Hp* strain [8]. Similarly, Cagδ and CagT were also found to mutually stabilise each other [34]. Therefore, we tested the stability of CagE in various deletion mutants of *cag*-PAI genes essential for CagA translocation. Cell extracts were prepared from different isogenic deletion mutants (mentioned on the figure) of *Hp* to test the stability of the protein. Next, equal amount of extracts from individual mutant strain was separated in SDS-PAGE and Western blotted using anti-CagE and anti-OMP antibodies (loading control). As shown in Fig 2A, CagE was detected at the wild-type level in all the mutant strains tested.

### ATPase activity of CagE

VirB4 proteins are a family of conserved ATPases present in almost all the reported T4SS [35]. In the published literature at least 38 VirB4 homologues have been reported till date [15]. In *Hp*, four VirB4 homologues are reported including CagE (HP0017, HP0441, HP0459 and HP0544) [36]. CagE is not only a VirB4 homologue but also the largest at 983 amino acids. However, till date nothing is known except its sequence analysis and requirement in Cag-T4SS function [21,37]. The sequence analysis revealed that like prototypical VirB4 of *Ag*, CTD of CagE contains conserved Walker A and Walker B motifs [21]. Until recently, no ATPase activity has been experimentally demonstrated for any of the VirB4 homologues of the protein translocating T4SS. However, recently ATPase activities have been shown for two VirB4 homologues TraB and TrwK of the conjugative plasmids pKM101 and pR388 from *E*. *coli* [14,15].

Therefore, to investigate the ATPase activity of CagE, we first cloned the full length gene and then tried to express it but failed to get expression. In an alternative approach we cloned the conserved CTD and first 531 amino acid stretch of N-terminal of CagE with GST tag. The clones were expressed in *E*. *coli* and respective recombinant proteins were purified following published protocol. The expressed recombinant proteins are named as CagEC (541–983, aa; C-terminal) and CagEN (1–531, aa; N-terminal) respectively and shown in S1A and S1B Fig. To determine whether the expressed CagE peptides exhibit ATPase activities, the release of inorganic phosphate (Pi) from [γ-^32^ P] ATP was monitored in the presence of GST-CagEC (541–983, aa), GST-CagEN (1–531, aa) and GST as a control. GST-CagEC (541–983, aa) and GST-CagEN (1–531, aa) recombinant proteins tested exhibited concentration and time-dependent ATPase activities, whereas the control GST protein did not show any such activity under the same experimental conditions (Fig 2B and 2C, S2A and S2B Fig). The mutant form of the GST-CagEC (541–983, aa), containing a point mutation in the ATP binding domain (K to A within the ‘GSTGSGKT’ NTP binding motif) exhibited no ATPase activity when compared to wild-type protein (S2C Fig). It is worth mentioning that Durand et al., recently analysed N-terminal segments of a number of VirB4 homologues and reported presence of degenerated Walker A like nucleotide binding site, but not in CagE [15]. Rabel et al., also did not observe any such degenerated nucleotide binding site in their analysis of CagE [21]. The ATPase activity observed in GST-CagEN is, however, surprising and we have no explanation to offer. In future further studies need to be done to understand this dilemma. We performed an immune-depletion experiment using CagE-specific antibody to test specificity of the reactions. As shown in S2D and S2E Fig, immune-depleted samples exhibited no ATPase activity compared to samples where control IgG were used, demonstrating that these ATPase activities are specific to the proteins under study rather than arising from possible contaminants in the purified samples.

### CagE interacts with the inner membrane proteins Cagβ and CagV

**Fig 3:**
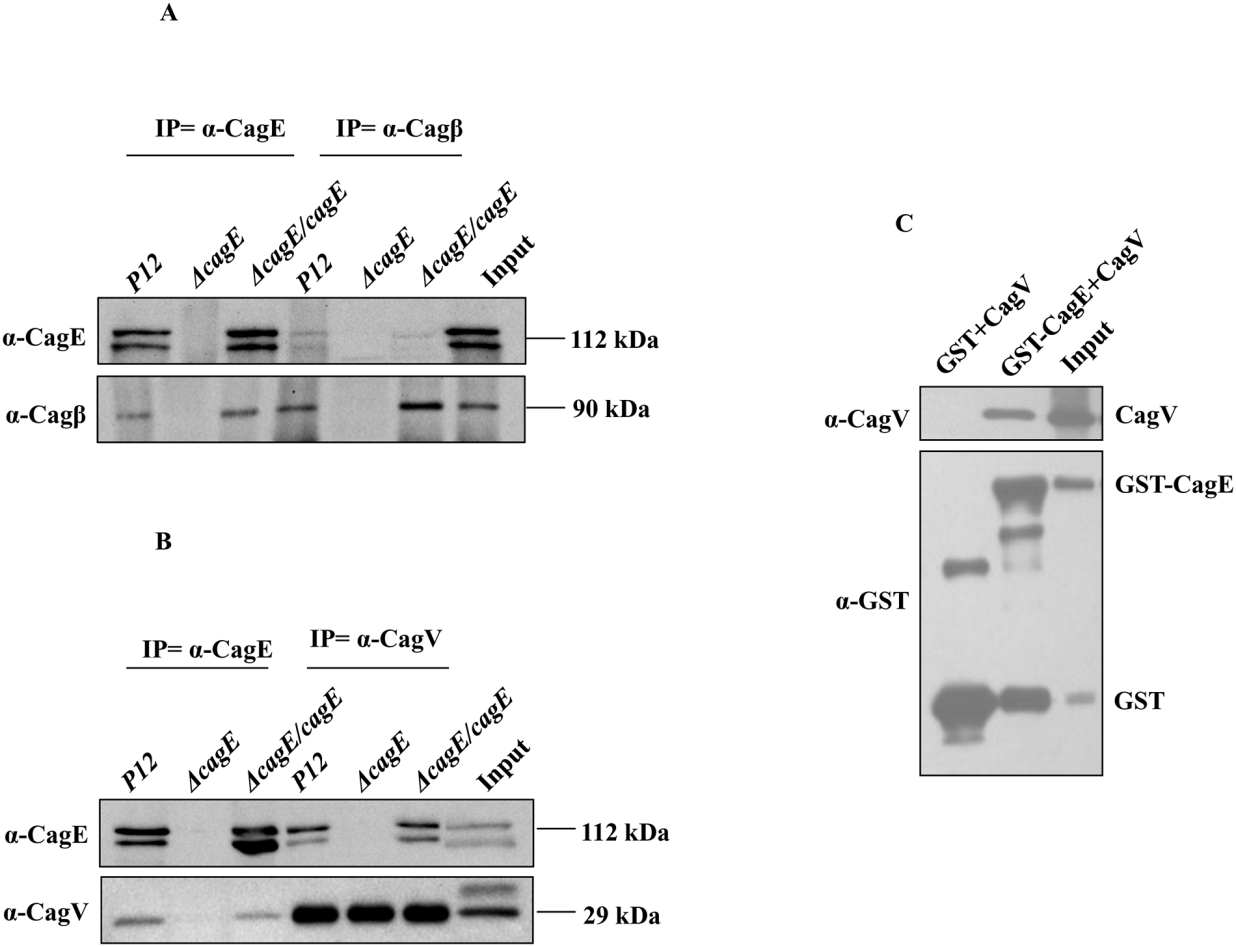
CagE interacts with Cagβ and CagV. **(A)** Western blots showing co-immunoprecipitation (Co-IP) of Cagβ and CagE from cell extracts prepared from wild-type *Hp (P12)* and *P12ΔcagE/cagE* by anti-CagE and anti-Cagβ antibodies respectively. *P12ΔcagE* strain was used as a negative control. Antibodies used in the Western blots are marked. **(B)** Western blots showing Co-IP of CagV and CagE from cell extracts prepared from wild-type *Hp (P12)* and *P12ΔcagE/cagE* by anti-CagE and anti-CagV antibodies respectively. *P12ΔcagE* strain was used as a negative control. Antibodies used in the Western blots are marked. **(C)** Western blots showing GST-pull-down of CagV by GST-CagE. Antibodies used in Western blotting and precipitated proteins are indicated.

Since CagE is shown above to be an inner membrane associated energy providing component, it might interact with other inner membrane associated Cag components like prototypical VirB4 [38]. To test which of the inner membrane proteins interact with CagE, co-immunoprecipitation (Co-IP) was performed on TritonX-100 solubilised wild-type *Hp* (*P12*), *P12*Δ*cagE*, *P12*Δ*cagE/cagE* cell extracts using anti-CagE, anti-CagV and anti-Cagβ antibodies as described in materials and methods. Co-immunoprecipitated samples were separated in SDS-PAGE and Western blotted using the inner membrane proteins specific antibodies, i.e., anti-CagE, anti-CagV, anti-CagF, anti-Cagβ, and anti-CagZ antibodies. As shown in Fig 3A and 3B, the anti-CagE antibody was found to co-immunoprecipitate only Cagβ and CagV. However, no protein signals corresponding to CagV and Cagβ were observed where *P12*Δ*cagE* extracts and anti-CagE antibody were used. Likewise, the same result was obtained when the anti-CagV and anti-Cagβ antibodies were used in Co-IP (Fig 3A and 3B). When extracts from *cagE* complemented strain *P12*Δ*cagE/cagE* was used the wild-type results re-appeared (Fig 3A and 3B). Taken together, these results confirm the interactions of CagE with CagV and Cagβ under physiological condition. However, these results do not indicate whether these interactions are direct or require accessory factor(s). Next, we performed GST pull-down assay on bacterial extracts prepared from co-expressed recombinant GST-CagE and CagV proteins and demonstrated that the interaction between CagE and CagV is direct (Fig 3C). Interaction of CagE with inner membrane components CagV and Cagβ was also performed in *Hp 26695* background and we found the same result (data not shown).

### CagE is required for the localisation/stability of CagI, CagL and CagH

Being a VirB4 homologue, CagE is expected to have role in the Cag-T4SS pilus formation. Prototypical VirB4 and its homologues from conjugative systems are predicted to have roles in pilus biogenesis [39,40]. Therefore, we tested the role of CagE in pilus biogenesis by TEM (transmission electron microscopy) and SEM (scanning electron microscopy) on wild-type *Hp26695*, and *Hp26695ΔcagE* strains in pure culture and under infection conditions respectively. We observed that deletion of *cagE* affects pilus synthesis (S3 Fig). Schaffer et al., also reported similar effect [39].

**Fig 4:**
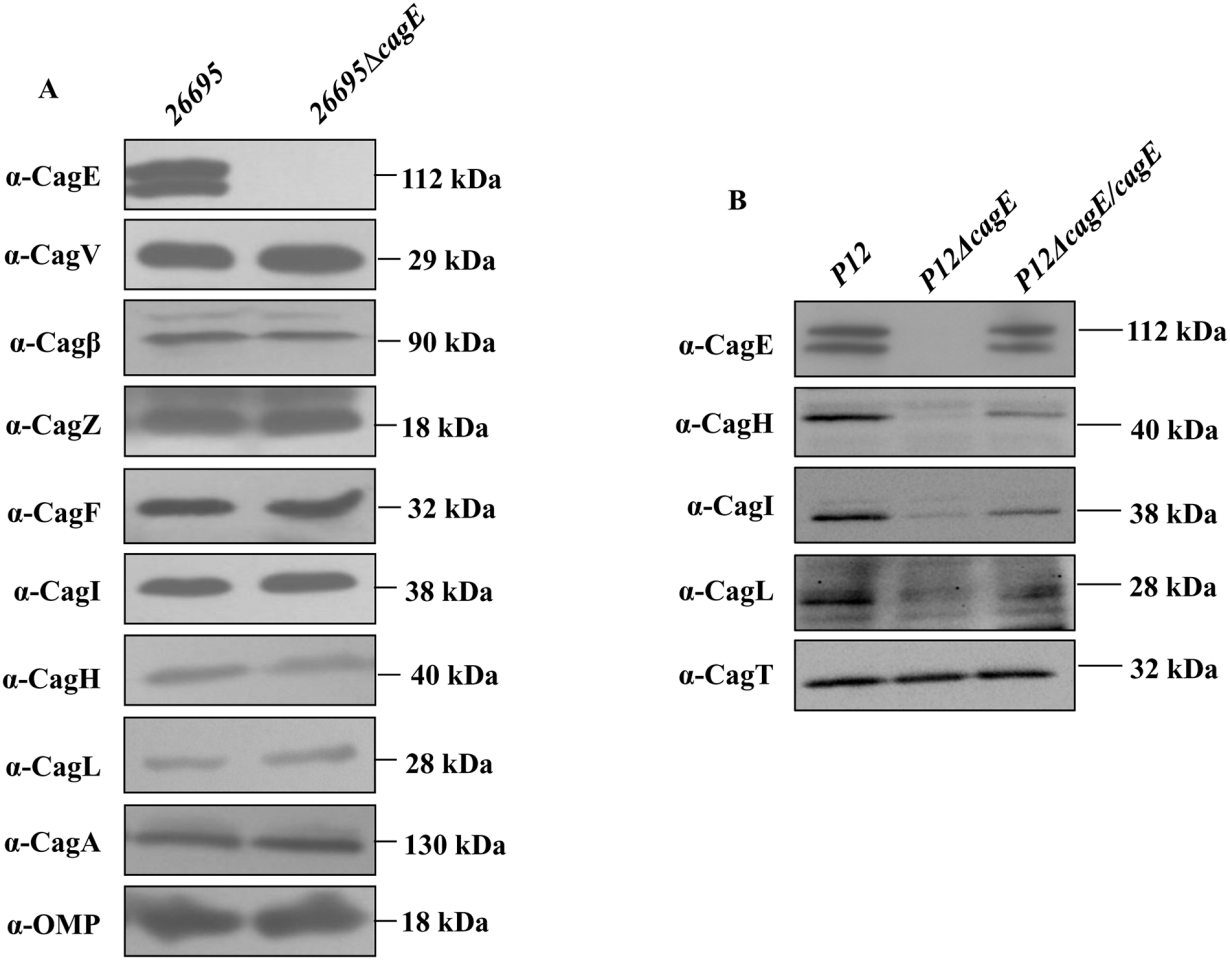
(A) Western blots showing stability of inner membrane and pilus associated Cag proteins in the absence of CagE in *26695ΔcagE*. Antibodies used in Western blots are indicated. CagA and OMP are used as loading controls. **(B)** Western blots showing stability of pilus associated CagH, CagI and CagL in wild-type *P12*, *P12ΔcagE* and *P12ΔcagE/cagE*. CagT was used as a loading control. Antibodies used in Western blotting are indicated.

**Fig 5:**
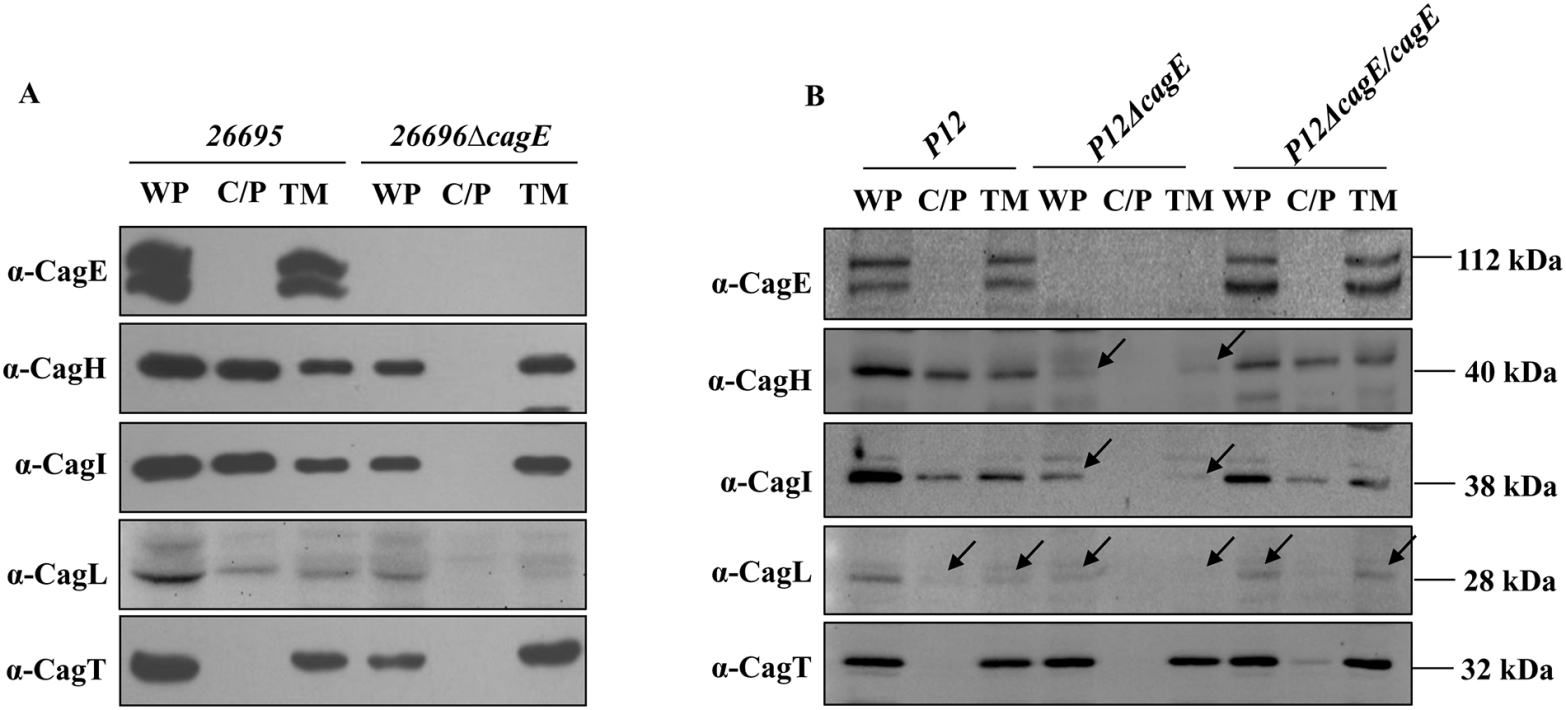
(A) Western blots showing sub-cellular localisation of pilus associated Cag proteins (CagH, CagI, and CagL) in wild-type and isogenic *cagE* mutant. WT, C/P and TM indicate whole cell lysate, soluble cytoplasmic/periplasmic and total membrane fractions respectively. Antibodies used in Western blots are indicated. CagT was used as membrane protein control. **(B)** Western blots showing sub-cellular localisation of CagH, CagI, and CagL in wild-type *P12*, *P12ΔcagE* and *P12ΔcagE/cagE* strains. WT, C/P and TM are as in panel **A**. Antibodies used are marked.

We therefore asked the question how CagE affects pilus formation. To search for answers, we first looked into the stability and localisation of known Cag-T4SS inner membrane proteins CagV, Cagβ, CagZ, CagF and pilus associated proteins CagI, CagL and its predicted regulator CagH in the absence of *cagE* in *Hp26695ΔcagE* and *P12ΔcagE* strains [39,41]. Cell extracts from wild-type *26695*, *P12*, mutants *26695ΔcagE*, *P12ΔcagE*, and *cagE* complemented *P12ΔcagE/cagE* strains were prepared, separated in SDS-PAGE and subjected to the Western blot analysis using the indicated antibodies. As shown in Fig 4A, the stability of none of the proteins were affected in the absence of CagE, except that of Cagβ in *26695ΔcagE*. Stability of Cagβ is slightly reduced compared to the wild-type strain. However, unlike in *26695ΔcagE*, stability of CagI, CagL and CagH were found to be affected in *P12ΔcagE* strain (Fig 4B). Nonetheless, complementation of the wild-type function restored back the native status in *P12ΔcagE/cagE* (Fig 4B). *P12*Δ*cagE* deletion mutant strain was employed here to overcome certain technical difficulties associated with the strain *26695* in gene complementation studies. It is worth mentioning at this point that *Hp* strain *26695* has unusually very low gene complementation ability for certain sets of gene compared to strain like *P12* [32]. CagA and OMP were used as loading controls for the stability studies in *26695*, while CagT was used as loading control in *P12* studies. Next, we looked into the localisation of CagI, CagL and CagH in the mutant strains *26695ΔcagE* and *P12ΔcagE* and *cagE* complemented strain *P12ΔcagE/cagE* by cell fractionation assay. As shown in Fig 5A, pilus associated CagI, CagL and surface exposed CagH were recovered in the membrane fraction only from *26695*Δ*cagE* mutant strain. On the other hand in wild-type cell extracts these proteins were detected in both the soluble and membrane fractions. Although these proteins were found to be unstable in *P12ΔcagE*, still the residual proteins were detected only in the membrane fraction **(** Fig 5B, **see arrow)**. Nonetheless, when the mutant gene was complemented with wild-type *cagE* allele in *P12*Δ*cagE/cagE* strain the wild-type status was restored back (Fig 5B). These results therefore suggest that absence of CagE made these proteins mis-localised in *26695ΔcagE* while unstable and mis-localised in *P12ΔcagE*.

### CagE is required for CagA translocation and IL-8 induction

**Fig 6:**
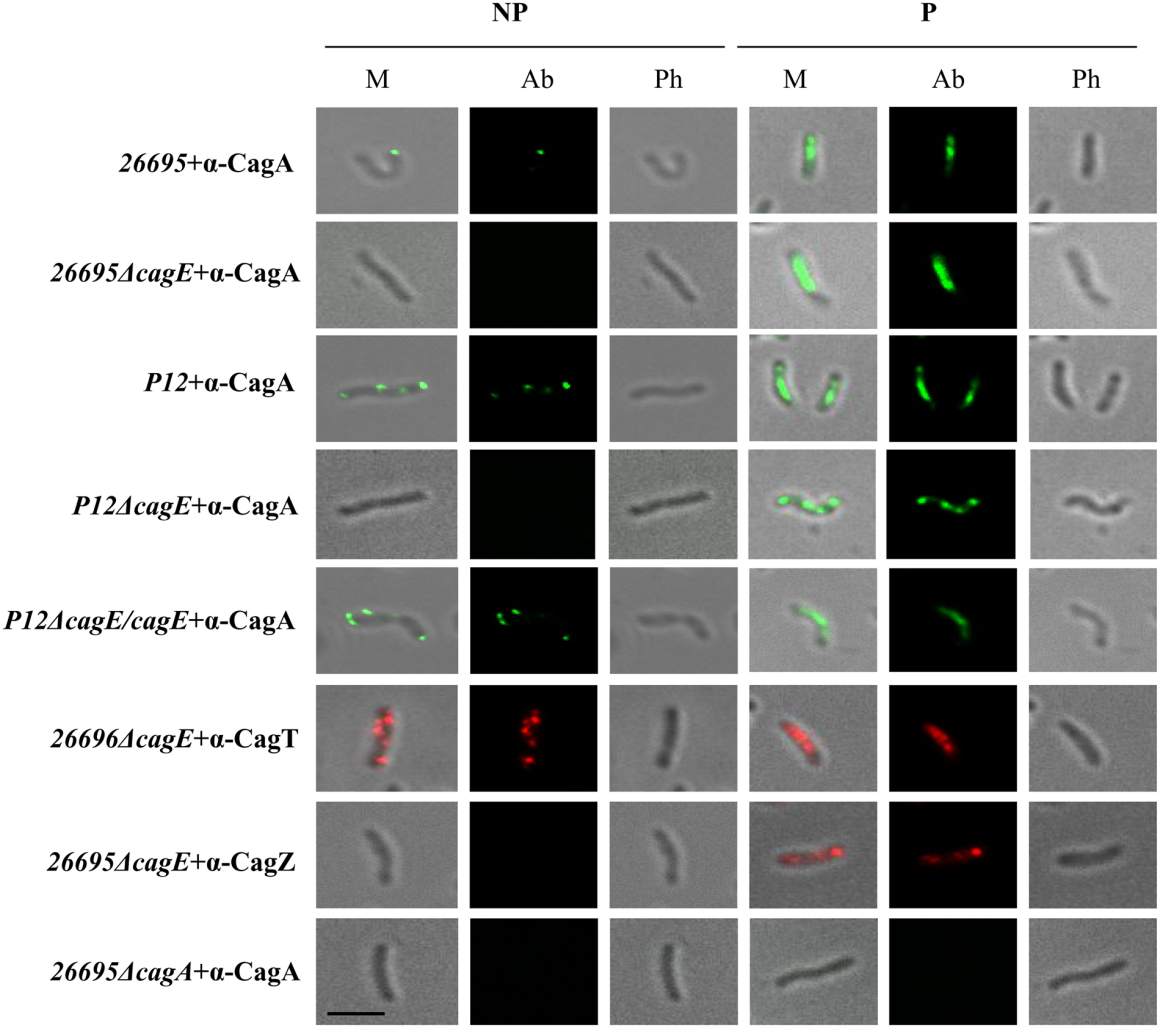
IFM showing CagE dependant surface localisation of CagA in wild-type *Hp 26695* and *P12* cells. *26695ΔcagA* and *P12ΔcagE* strains were used as negative controls. Localisation of CagT and CagZ in *26695ΔcagE* strains were used as controls for surface and inner membrane localised proteins respectively. NP and P stand for non-permeabilised and permeabilised cells respectively. M, Ab, and Ph stand for Merge, respective antibody used, and Phase respectively. Primary antibodies used are indicated. Alexa fluor 488 (green) and Alexa fluor 594 (red) conjugated secondary antibodies were used for immuno detection. Scale bar indicates 5 μm.

**Fig 7:**
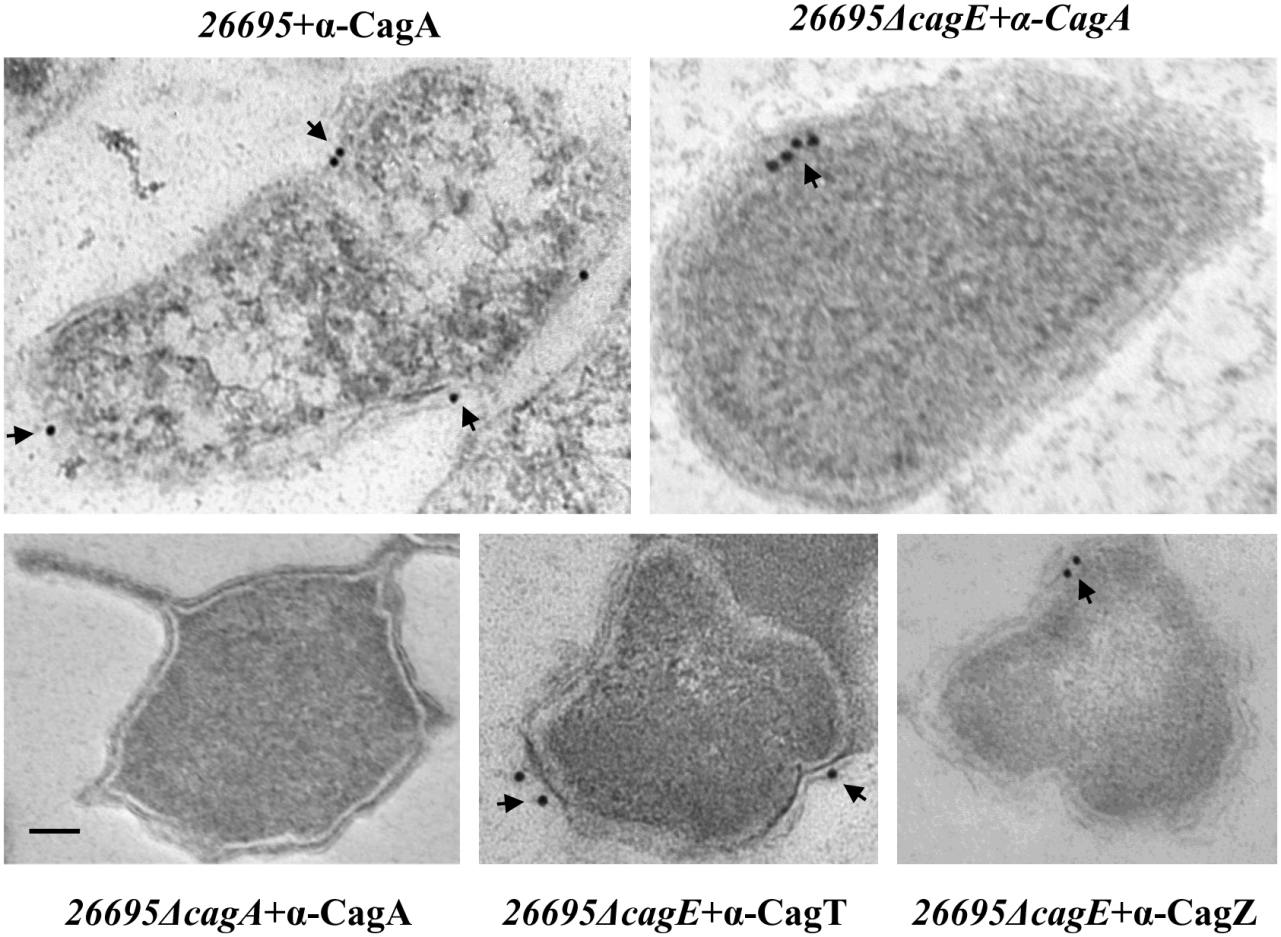
Immunogold electron microscopy showing localisation of CagA in wild-type and *26695ΔcagE* strains. Ultra thin sections of wild-type, *26695ΔcagE* and *26695ΔcagA* mutant cells were immunostained with anti-CagA and gold-labelled secondary antibody. *26695ΔcagA* strain was used as a negative control for CagA antibody. Localisation of CagT and CagZ were used as a control for surface exposed and inner membrane proteins. Scale bar indicates 100 nm. Arrowheads indicate location of gold-labelled secondary antibody.

Earlier it was reported that the VirB4 homologue in *Hp*, CagE is required for CagA translocation through Cag-T4SS and also needed for the secretion of IL-8 by the host gastric epithelial cells [37]. The prototypical VirB4 in *Ag* is also known to require for substrate translocation and pilus formation [40,42]. We therefore wanted to test its substrate translocation function and IL-8 induction in isogenic *cagE* deletion mutant strains *26695ΔcagE* and *P12*Δ*cagE* respectively in our hand. In this regard, we first studied CagA surface localisation on isogenic *26695*Δ*cagE* and *P12*Δ*cagE* strains by IFM. Wild-type strains *26695* and *P12* were used as a positive control. As shown in Fig 6, unlike wild-type *26695* and *P12* strains no CagA specific signal was detected on *26695*Δ*cagE* and *P12*Δ*cagE* strains under non-permeabilised condition. However, when *cagE* null function in *P12*Δ*cagE* was complemented with wild-type *cagE* allele in *P12*Δ*cagE/cagE* CagA signal was re-appeared on the bacterial cell surface under non-permeabilised condition (Fig 6). Similarly CagT and CagZ specific signals which do not depend on *cagE* function were also visualised under similar condition as controls for surface exposed protein and inner membrane protein respectively [31,32]. Further, we also studied surface localisation of CagA by TEM in wild-type *26695* and *26695ΔcagE* strains. Unlike in wild-type strain, CagA specific signal was found to be absent on the cell surface in *cagE* deficient strain as reported earlier (Fig 7) [41,43]. CagT and CagZ specific signals were used as controls for outer and inner membrane proteins respectively [31,32]. As mentioned previously we employed *P12*Δ*cagE* strain instead of *26695*Δ*cagE* for better transformability of the former strain [32]. To complement the *cagE* null function another *Hp* strain was constructed where a wild-type *cagE* gene under *cagA* promoter was inserted into the *P12*Δ*cagE* chromosome in *recA* locus [32].

Next, to study the Cag-T4SS function in the absence of *cagE* or in the *cagE* complemented strain, we infected gastric epithelial cells (AGS) with wild-type *P12*, *P12*Δ*cagE*, and *P12*Δ*cagE*/*cagE* strains and analysed CagA phosphorylation, IL-8 secretion and induction of humming bird phenotype that are associated with an active Cag-T4SS. As shown in S4A Fig, *cagE* mutant strain was unable to translocate CagA as measured by its phosphorylation. The deletion strain was also unable to induce IL-8 secretion by gastric epithelial cells (S4B Fig). It was also failed to induce humming bird phenotype that was associated with active Cag-T4SS function as reported earlier [37]. However, all these functions were restored back in the *cagE* complemented *P12*Δ*cagE/cagE* strain (S4A and S4B Fig). Hence, this study reconfirms the involvement of CagE in CagA translocation and IL-8 induction by *Hp* Cag-T4SS.

### Comparative sequence analysis, homology modeling and molecular dynamics simulations of CTD of CagE

Our in-vitro study, demonstrated ATPase activity of CTD of CagE (541–983, aa). In one of the previous study, Rabel et al., showed the presence of C-terminal conserved motifs in eighteen different VirB4 homologues including CagE [21]. These motifs were essential for conjugation and phase adsorption and play important role in macromolecular transport across the membrane [21]. To further elucidate the extent of conservation in CagE, we performed a multiple sequence alignment of ten different homologues of VirB4 from various bacterial species including CagE. We also observed the same conserved motifs A (Walker A), B (Walker B), C, and D among these ten homologues (S5 Fig). The percent identity of CagE with other VirB4 homologues is shown in S1 Table.

Recently, crystal structure of CTD (residue covering 205 to 588, aa) of tpsVirB4 (PDB ID: 4AG5) has been published and it was found to be very similar to the structure of CTD of TrwB (RMSD value 3.5 Å). However, these homologues share a sequence identity of just 12% over the structurally aligned residues [19]. This suggests that sequence identities among these proteins are low but they share similar folds and structure. Based on this information we modelled CTD of CagE (residue covering 541 to 965, aa) through homology modelling and performed a 60 ns molecular dynamics simulation to obtain its stable conformation. Over 60 ns simulation time, with respect to starting conformation, new conformations have higher RMSD and lower Radius of gyration around axis which converges after 45 ns (S6A and S6B Fig). The number of intra-protein hydrogen bonds has also increased relatively from the starting point and converge after 35 ns (S6C Fig). Looking at residue wise RMSF, fluctuations were observed across the whole protein, and in the last 40 ns observed fluctuations were relatively lower than in the first 20 ns of the run (S6D Fig). These results demonstrate that through simulations we obtained a stable conformation of CagE CTD with relatively higher compactness and less fluctuation.

**Fig 8:**
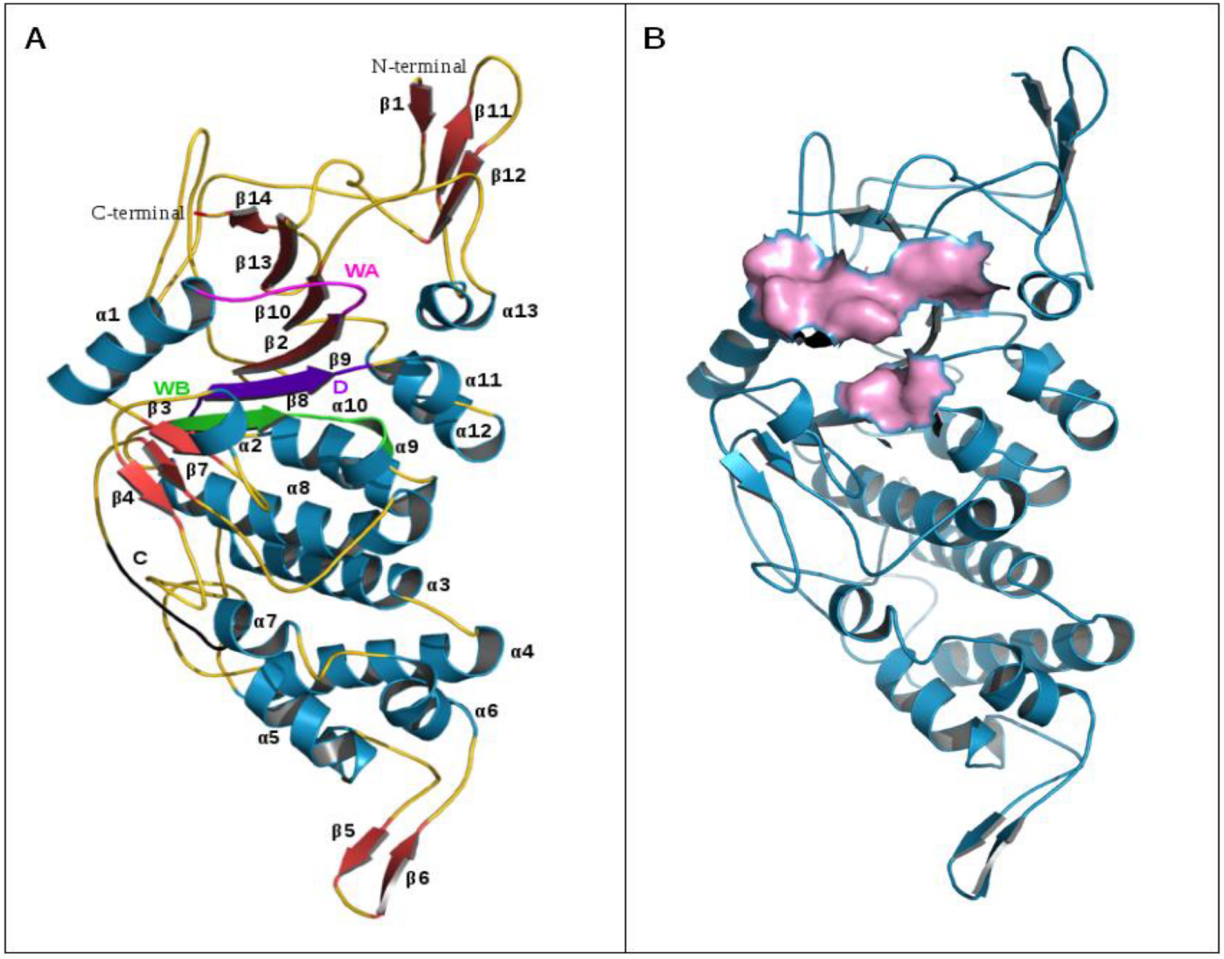
Structure of CTD of CagE. **(A)** Structure of CagE (residue cover: 541 to 965, aa). α-helices (α1 to α13) are numbered and shown in dark cyan, β sheets (β1 to β14) are shown in brick red and coils are shown in gold. Motif A (Walker A), motif B (Walker B), motif C and motif D are shown in magenta, green, violet and black respectively. **(B)** ATP binding site of CagE, (Walker A and Walker B) shown in surface view (Pink).

Overall structure of CagE CTD was almost similar to CTD of tpsVirB4 and TrwB and comprised of 13 α-helices and 14 β-strands (Fig 8A). Unlike tpsVirB4 and TrwB structures it cannot be subdivided explicitly into two domains (i.e. the α/β domain and helical bundle domain) as an additional anti-parallel β-sheet comprising two β-strands (β5 and β6) were formed in the region corresponding to helical bundle of tpsVirB4 and TrwB. α/β domain which is Rec-A like, contains Walker A, Walker B, motif C, and D was more structurally conserved. The residues involved in ATP binding were reported for both the templates [19,44]. Through structure alignment, we identified the conserved corresponding residues in CagE. These residues belong to the highly conserved motif A (Walker A) and motif B (Walker B) and are in close proximity with each other at structure level (Fig 8B). Therefore, it is highly probable that conserved residues Gly600, Gly602, Lys603, Val605, Asp830, and Glu831 of CagE participate in ATP binding and hydrolysis, however, its further validation is required.

## Discussion

With an objective to understand the biogenesis and substrate translocation mechanism of the Cag-T4SS, we have characterised the putative VirB4 homologue CagE, one of the energy providing components of *Hp*. We report here that like other VirB4 homologues it is an inner membrane associated protein and its stability does not depend on any other Cag components tested in this study (Figs 1 and 2A). Prototypical VirB4 in *Ag* is, however, unstable in the absence of VirB8 [33]. In the absence of *cagE* in *26695*Δ*cagE*, stability of only Cagβ, a VirD4 homologue was slightly affected but not of any other Cag proteins (Fig 4A). Nevertheless, in *cagE* deficient *P12* strain destabilisation of CagI, CagL and CagH is observed (Fig 4B).

In contrast to the prototypical VirB4, which interacts with both the energy providing components VirB11 and VirD4, CagE interacts with Cagβ only but not with Cagα (a VirB11 homologue) (Fig 3A) [42]. VirB4 is also known to interact with a number of VirB components, notably VirB8, VirB6, and VirB10 [22–24]. Similarly, CagE interacts with VirB8 homologue CagV but not with CagZ and CagF that are unique Cag components present in the inner membrane (Fig 3B). Cagβ on the other hand interacts with CagZ [32]. Cagβ is also reported to interact with CagA, the Cag-T4SS substrate [32]. CagF an inner membrane protein also interacts with CagA [29,30]. These interaction studies were performed either by IP, Y-2H (yeast two hybrid) or GST-pull-down experiments [32]. Thus, CagE might be a part of large Cag complex at the inner membrane gate as all these are inner membrane associated proteins [29,32]. It seems that CagA is transferred to the secretion system through this inner membrane gate complex. Ideally, anti-CagE antibody should have immunoprecipitated all these proteins. However, the experimental result did not reflect the same. One of the explanations for this could be that during antigen-antibody interaction some kind of conformational change occurred in the protein complex leading to dissociation of components weakly attached to the core complex. Nonetheless, we are not ruling out other possibilities. Our study has also detected interaction of CagE with the outer membrane sub-complex components through CagV **(data not shown**, S7 Fig **)**.

The most important finding of the study is the concentration and time dependent ATPase activity of the CagE (Fig 2B and 2C, S2A and S2B Fig). In literature, ATPase activity of any VirB4 homologue is not experimentally demonstrated except in two conjugative plasmid’s VirB4 homologues TraB and TrwK [14,15]. Unlike the conjugative plasmid’s counterpart TraB, we observed ATPase activity in NaCl containing buffer rather than acetate buffer as reported by Durand et al., [14,15]. However, we have not tested oligomerisation status of the soluble peptides that show the enzyme activity. Both TraB and TrwK VirB4 homologues form hexamer in the acetate ion containing buffer and exhibit ATPase activity [14,15].

Recently Wallden et al., reported the crystal structure of tpsVirB4 and found that the structure closely resembles to that of TrwB which has low over all sequence identity to tpsVirB4 [19]. Therefore, we modelled the CTD of CagE and obtained its stable conformation through molecular dynamics simulations (Fig 8A). The obtained structure is almost similar to that of the crystal structure of its templates tpsVirB4 CTD and TrwB with major variation observed in region corresponding to helical bundle (Fig 8A). The functionally important Rec-A like α/β domain, known to be involved in ATP binding and hydrolysis is more structurally conserved among these three homologues. The residues Gly600, Gly602, Lys603, Val605, Asp830, and Glu831 of CagE from motif A (Walker A) and motif B (Walker B) may be involved in ATP binding and hydrolysis and forms the active site of CTD of CagE. Despite in the variation of size among these proteins and differences in their biological functions, all contain a conserved motor domain (α/β domain) that indicates these proteins are evolved from a common ancestor.

Durand et al., following *in silico* analysis of a number of VirB4 homologues including two from *Hp 26695* (but not CagE) identified degenerated nucleotide binding sites in the N-terminal half of the proteins [15]. Only three of these VirB4 homologues found to share the NBD2 Sec-A like motif and in one of them, TraB from pKM101, they experimentally demonstrated nucleotide binding and ATP hydrolysis and define it as a new class of VirB4 protein [15]. While analysing the C-terminally located conserved ATPase domain of CagE, we also analysed the N-terminal half of the protein experimentally and surprisingly observed ATPase activity (Fig 2B and 2C, S2A and S2B Fig). The activities are indisputable since immune-depleted samples exhibit no ATPase activity (S2D and S2E Fig). However, we do not have any explanation how a sequence having no defined/known nucleotide binding/hydrolysis motif exhibits ATPase activity. Nonetheless, this surprising result may in future lead to experimental study of the N-terminal half of the remaining VirB4 homologues for ATPase activity and their contribution to the respective system.

VirB4 and its homologues are known to participate directly in substrate translocation and pilus biogenesis [17,42]. Mutation in energy generating Walker A motif is known to affect the substrate translocation process but not the latter [42]. Here we have demonstrated that CagA translocation across the bacterial envelope to the cell surface and into the host epithelial cells is also dependent on CagE as the substrate transportation has stopped at the inner membrane (Figs 6 and 7, S4A Fig). We postulate that in the absence of required energy from CagE the substrate could not be transported into the trans-membrane channel and thus it gets stuck at the inner membrane gate as the native function is restored when *cagE* was supplied back (Fig 6 and S4A Fig).

Induction of IL-8 secretion, a major function of Cag-T4SS is also CagE dependent (S4B Fig). Role of ATPase activity on the above processes, however, could not be established. Not much is known about pilus biogenesis in *Hp* except a few recent reports [8,39,45,46]. In *Hp* prototypical pilus subunits are missing. Instead, topological analogue of VirB2, CagC and a weak VirB3 like sequence at the N-terminus of CagE have been predicted [8]. In fact in our immunoblot analysis of the CagE we have observed two closely paced bands; one of them could be processed (Fig 1A). These protein bands are specific as they reappear following complementation of *cagE* function. *Hp* also adapted a VirB5 like adhesion protein CagL that is shown to interacts with the host receptor β-integrin [8,43]. Recently few groups have reported involvement of CagH, CagI, and CagL in Cag-T4SS pilus formation [39,41]. Here, we have reported that pilus biogenesis is affected in the absence of CagE (S3 Fig). Schaffer et al., also reported requirement of CagE in Cag-T4SS pilus formation [39]. In *Ag* it is known that VirB4 dislodges the pilin subunit VirB2 from the membrane pool and triggers pilus formation [40]. Although CagC is a predicted VirB2 analogue its stability and localisation status are not known in the absence of CagE. Due to non-availability of specific antibody against CagC we could not test it. Nonetheless, we made an interesting observation that unlike in wild-type *Hp*, in the absence of CagE, CagI, CagL the pilus associated proteins and CagH (a predicted regulator of pilus biogenesis) are associated with the membrane (Fig 5A). Although, we are not sure about the mechanism of CagE involvement, we speculate that it might have something to do with the arrests of CagI, CagL and CagH in the membrane in its absence (in *26695*Δ*cagE*). However, we are not ruling out other possibilities. When the experiment was re-performed in *P12ΔcagE* mutant strain we found them very unstable compared to wild-type *P12* but the remaining residues were again detected in the membrane fraction like in *26695ΔcagE* **(** Fig 5A and 5B, **see arrow)**. However, when the *cagE* null function was restored back in the complemented strain *P12ΔcagE/cagE* all these proteins regain their location and stability like wild-type strain. Suggesting a major role of CagE (may be including associated VirB3 function) in the localisation and stability of Cag-T4SS pilus associated components and thus in pilus biogenesis. Further work is needed to decipher the mechanism.

## Materials and Methods

### Bacterial strains and growth conditions

*Hp* wild-type (*26695*, *P12)* and mutant strains were grown on 3.7% w/v brain heart infusion (BHI) agar (Difco) supplemented with 7% foetal calf serum (FCS), 0.4% campylobacter growth supplement and *Hp* dent supplement (Oxoid). *Hp* wild-type, mutant strains and *E*. *coli* strains used in this study are listed in S2 Table. Culture plates were incubated at 37°C for 24–36 hr in a GasPak anaerobic system using GasPak EZ sachet (BBL). *Hp* mutant strains were selected on BHI serum plates supplemented with chloramphenicol (6 μg/ml). *Hp* wild-type and mutant strains were maintained as frozen stocks at -70°C in 70% brain heart infusion media supplemented with 20% glycerol and 10% FCS. *E*. *coli* strains DH5α and BL-21 (DE3) were grown in Luria Broth (LB) or on LB agar plates supplemented with ampicillin (100 μg/ml), kanamycin (50 μg/ml), or chloramphenicol (35 μg/ml) to amplify plasmid DNA or to express recombinant proteins as appropriate.

### Cloning of *cagE* domains

DNA sequences that code for N-terminal (1–531, aa), and C-terminal (541–983, aa) of CagE were PCR amplified from genomic DNA of *Hp* strain *26695* and cloned into pGEX-6P-2 vector between BamHI and SalI restriction enzyme sites having N-terminal GST tag. All the cloned fragments were verified by DNA sequencing. Recombinant plasmids were transformed into competent *E*. *coli* BL21 (DE3) cells for large scale production of the respective proteins. Walker A box mutation was introduced into the plasmid carrying the C-terminal sequence of CagE, CagEC (541–983, aa). The point mutation was generated using the primer pair’s fmutCagE/rmutCagE as shown in S3 Table and their sequences are given in S4 Table. These primers mutate Lys603 in the Walker A motif to Ala603. Following the PCR amplification, the product was digested with DpnI to remove template DNA, purified, and was transformed into *E*. *coli* BL21 (DE3) cells. The presence of mutation in the recombinant plasmid was verified by sequencing.

### Production and purification of recombinant proteins

*E*. *coli* BL21 (DE3) cells harbouring recombinant plasmid encoding GST-CagEN (1–531, aa), or GST-CagEC (541–983, aa) or Walker A mutant were grown at 37°C in LB media supplemented with 100 μg/ml ampicillin till OD_600nm_ reached a value of 1. Cultures were cooled to 20°C, IPTG (isopropyl-β-D-thiogalactopyranoside) was added to a final concentration of 0.1 mM, and growth was continued for 16 hr at 20°C. Cells were harvested by centrifugation and stored at -70°C. Next, the cells were thawed, re-suspended in 1X PBS containing 1% TritonX-100, 1 mM PMSF, 2 mM DTT, 1 mM EDTA, and 1 mg/ml lysozyme, mixed well and incubated at 4°C for 45 min. Cells were then ruptured by sonication, and lysate was clarified by centrifugation at 18000 rpm for 30 min in a JA-20 rotor in a Beckman Coulter centrifuge. The clarified lysate was then used to bind with pre-equilibrated glutathione beads for 2 hr. After binding beads were washed three times with wash buffer (1X PBS, 1 mM PMSF, 2 mM DTT, 1% TritonX-100, 300 mM NaCl) and finally bound proteins were eluted in elution buffer (50 mM Tris-HCl, pH 8.0, 150 mM NaCl, 0.05% TritonX-100, 10 mM DTT, 10% glycerol, and 20 mM glutathione), aliquots were made and stored at -70°C for further use. All the purification steps were carried out at 4°C.

### ATPase activity of the CagE domains

The ATPase activities of the CagE NTD, and CTD were measured individually in 10 μl reaction mixtures containing 20 mM Tris-HCl (pH 8.0) 1 mM MgCl_2_, 20 mM KCl, 8 mM DTT, 4% sucrose, 16 μg/ml BSA, 1 mM ATP, 3.4 fmol of [γ-^32^P] ATP and the required amount of the CagE domains, as indicated on the figure. The reaction mixtures were incubated at 37°C for 90 min, and the reactions were stopped by placing the tubes on ice. The released inorganic phosphate (Pi) was separated by thin layer chromatography (TLC) on a polyethylene cellulose strip (Sigma-Aldrich) in 0.5 M LiCl and 1 M formic acid at room temperature for 1 hr. The TLC plate was dried and auto-radiographed.

### Immunodepletion assay

For immunodepletion assays, aliquots of the purified CagE domains were incubated with purified IgG from preimmune or anti-CagE antibodies at 4°C for 60 min. The antigen-antibody complexes were removed by adding protein-A agarose beads. The supernatant was then used to determine ATPase activity as described above.

### Construction of mutator plasmid, transformation and complementation

To create the *cagE* null mutant strain, the mutator plasmid pBS-*cag*8AΔ*cagE/CatGC* was constructed following a previously published protocol [37]. Briefly, the genomic region of the *cag*8A (*cagC*, *cagD*, *cagE*, and *cagG* orfs) sequence was PCR amplified from *Hp 26695* genomic DNA using f*cag*8AN/r*cag*8BK primer pairs (N & K = NotI and KpnI; f & r = forward and reverse primers, respectively) and Phusion DNA polymerase (NEB) and cloned into pBluescript between KpnI and NotI sites, yielding pBS*cag*8A [37]. Primer pairs and their sequences are given in S3 and S4 Tables. Next, the plasmid pBS*cag*8A was copied excluding the sequence encoding *cagE* by inverse PCR using f*cagE*B/r*cagE*X primer pairs (B and X stand for BamHI and XhoI, respectively) (S3 and S4 Tables). The inverse amplified PCR product was digested with BamHI and XhoI and ligated with the terminator-free *CatGC* cassette amplified from the pBS-*CAT* plasmid by PCR using the F*cat*X/R*catB* primer pairs (X and B stand for XhoI and BamHI, respectively) (S4 Table). *E*. *coli* DH5α competent cells were transformed with the ligated product and plated on LB agar. Positive clones were first selected on a chloramphenicol and ampicillin containing plate and then finally verified by the double digestion of the plasmids isolated from the drug resistant colonies. Plasmids isolated from positive clones were then introduced into wild-type *Hp 26695* and *P12* strains by natural transformation. For complementation of *cagE* function in *P12ΔcagE* mutant strain, 400 bp *cagA* promoter was amplified from *Hp 26695* genomic DNA as a template and then cloned into pJP99 vector between SalI and BamHI sites. Next, *cagE* gene was cloned downstream of *cagA* promoter in pJP99 vector by digesting the vector with BamHI and KpnI restriction enzymes, and transformed into *E*. *coli* cells. Plasmids isolated from positive clones were then used to transform *P12ΔcagE* cells. Positive clones were selected on kanamycin and chloramphenicol containing BHI agar plate.

### AGS cell infection, tyrosine phosphorylation and IL-8 secretion

About 5X10^5^ AGS cells were seeded on 6 well culture plates and grown for 24 hr before infection. Infection with *Hp* was performed at a MOI of 1:100 and continued for 4 hr. Following infection, cell supernatant was collected and IL-8 secretion was measured by sandwich ELISA. Briefly, 2 μg of mouse monoclonal anti-IL-8 antibody [sc-8427, IL-8 (B-2), Santa Cruz Biotechnology] was used to coat each well of ELISA plate (Corning) O/N at 4°C, washed three times with washing buffer (1X PBS containing 0.01% Tween-20), blocked by 3% BSA for 2 hr, washed three times with washing buffer, 100 μl of culture supernatant was added to each well and incubated for 2 hr at 4°C. Next, wells were washed three times with washing buffer, anti-IL-8 antibody was added again, allowed to bind the antigen, washed three times, anti-mice secondary antibody (Sigma) was added to each well at 1:5000 dilutions and incubated for 1 hr. Wells were then washed again three times, chromogenic color substrate (2,2’-Azino-bis(3-ethylbenzothiazoline-6-sulfonic acid, Sigma) was added, incubated for 20 min and developed colors were measured at OD_405 nm_ by an ELISA plate reader.

For detection of phosphorylated CagA, following 4 hr of infection, cells were washed twice with PBS containing 10 mM sodium orthovanadate and re-suspended in 100 μl of 2.5X SDS sample loading buffer (0.125 M Tris-HCl, pH 6.8, 5% SDS, 100 mM β-mecaptoethanol, 25% glycerol, and 0.1% bromophenol blue). 20 μl of cell lysate was loaded on SDS-PAGE and presence of phosphorylated CagA was detected by Western blotting using mouse monoclonal anti-phosphotyrosine antibody [sc-7020, p-Tyr, (PY99), Santa Cruz Biotechnology].

### Ethics statement

This study was approved by the Institutional Animal Ethics Committee-of Jawaharlal Nehru University. The Institutional Ethics Committee Code no: 23/2007 and 22/2012.

The animals (Balb/c mice female or New Zealand white rabbit female) were maintained at the Central Animal Facility of the Jawaharlal Nehru University as approved by the Institutional Animal Ethics Committee. After experimental procedures were over, the animals were maintained until their natural death, and every effort was made to minimise their suffering.

### Antibodies, SDS-PAGE and immunoblotting

Several polyclonal antibodies against *cag*-PAI components were used in this study (S8 Fig). Details of anti-CagF, anti-CagT, anti-CagM, anti-CagX, anti-CagI, anti-CagH, anti-CagZ, anti-OMP and anti-CagA antibodies have been described previously [41]. For detection of GST tagged protein HRP conjugated rabbit anti GST antibody was used (GENEI, cat#HP024). For generation of anti-CagV and anti-Cagβ antibodies, CagV without tag and His tagged Cagβ [ΔN170, N terminal 170 amino acids were deleted] were cloned in pET-28a vector (CagV) and pET-14b (Cagβ). Proteins were over expressed in BL-21 (DE3) cells as inclusion bodies, solubilised in PBS containing SDS, separated in SDS-PAGE and desired recombinant protein bands were cut out from the gel. Next, gel slices were individually pulverised, re-suspended in equal volume of PBS and Freund’s adjuvant and resultants were used to generate antibodies in rabbit and mice respectively. Specificities of anti-CagV (rabbit), anti-Cagβ (mice) and anti-CagE (mice) antibodies were shown in S8 Fig. To generate polyclonal antibodies against CagE in rabbit and mice, the *cagE* gene (ΔN140, N terminal 140 amino acids were deleted) was cloned in the pGEX-6P-2 vector between the BamHI and SalI sites and expressed as GST-tagged protein in *E*. *coli* strain BL-21 (DE3). Generated inclusion bodies containing GST tagged CagE was processed as before and used for antibody generation. SDS-PAGE and Western blotting were performed as previously described [37,41]. Horse radish peroxidase-conjugated anti-rabbit IgG (Bio-Rad) and anti-mice IgG (Sigma) were used to visualise bound primary antibodies.

### Sub-cellular fractionation

*Hp* cells were grown on BHI agar plates, collected, washed twice with PBS and re-suspended in 500 μl of 20 mM Tris-HCl, pH 8.0. Cell fractionation was performed as described earlier [41]. Briefly, re-suspended cells were sonicated, unbroken cells and debris were removed by centrifugation at 8000 X g for 10 min at 4°C. The supernatant was centrifuged at 148,000 X g for 1 hr at 4°C in a SW-55 rotor, Beckman coulter ultracentrifuge. The supernatant was a mixture of cytoplasmic/periplasmic fractions (C/P), and the pellet was considered to be the total membrane fraction (TM). Fractionated samples were dissolved in 2X SDS sample buffer, boiled and subjected to SDS-PAGE, followed by Western blotting using appropriate antibodies.

### Immunofluorescence microscopy (IFM)

Immunofluorescence microscopy (IFM) of *Hp* cells was performed as described earlier, with minor modifications [41]. *Hp* cells were fixed on sterile glass cover slips with 4% paraformaldehyde for 10 min at RT. Following fixation, cells were permeabilised with 0.2% TritonX-100, and cover slips were blocked in 5% bovine serum albumin (BSA) in 1X PBS for 30 min. The cells were then incubated with specific polyclonal antibodies at appropriate dilutions (anti-CagE-1:500, anti-CagT-1:1000, and anti-CagZ-1:1000, anti-CagA-1:700) at 4°C for 2 hr. Thereafter, fixed cells were washed three times with PBS and then incubated with Alexa fluor 488 conjugated goat anti rabbit and Alexa fluor 594 conjugated goat anti mouse secondary antibodies (Invitrogen) for 1 hr at RT as required. The cover slips were mounted with 20% glycerol on glass slides and visualised at 100X through a Carl Zeiss fluorescence microscope equipped with oil immersion objectives. Images were captured using an Axio Cam Hrm digital camera and analysed by Axio-vision-4.8 software. The images were processed using standard image processing techniques.

### Co-immunoprecipitation

*Hp 26695*, *P12*, *P12ΔcagE*, and *P12ΔcagE/cagE* cells (~100 μl packed cell volume) were re-suspended in 1 ml of lysis buffer (1X PBS pH 7.4, 2 mM EDTA, 2 mM DTT, 1% Triton X-100, 1 mM PMSF, 1mg/ml lysozyme and 6 μl of 100X protease inhibitor cocktail), lysed by sonication [three cycles (30 sec each at 2 min cooling interval) at amplitude 4], centrifuged at 13K rpm for 30 min, and supernatant was pre-cleared by adding pre-immune rabbit serum or mouse serum as required, along with protein-A agarose beads. 500 μl of pre-cleared samples (1.0 mg/ml total protein) were used in Co-IP. To each pre-cleared sample 3 μl of desired antibody was added (anti-CagE, anti-Cagβ, anti-CagV), incubated on rotating rocker O/N at 4°C. Next, 25 μl of packed volume of protein-A agarose beads were added to each sample, incubated for an additional 2 hr, centrifuged at 4000 X g, supernatant was discarded, and the beads were washed with lysis buffer without lysozyme. Bound proteins were released by boiling the beads in 2X SDS sample buffer and then subjected to SDS-PAGE followed by Western blotting using the desired antibody.

### Co-expression and GST pull-down assay

*cagV* was cloned into pACYC-duet1 and expressed along with GST tagged *cagE* in pGEX-6P-2 in *E*. *coli* following a previously published protocol with minor modifications [47]. Primer pairs used in DNA amplifications and their sequences are given in S3 and S4 Tables. Transformed *E*. *coli* cells were selected on chloramphenicol/ampicillin double antibiotic-containing LB-agar. For GST pull down experiment, GST and recombinant CagV or GST-CagE and CagV were mixed in binding buffer (1X PBS, 1% TritonX-100, 1 mM DTT, 1 mM EDTA, and 1 mM PMSF), incubated with GST sepharose beads and protein bound beads were collected by centrifugation, washed 5 times with binding buffer, proteins were eluted by boiling in 2X SDS sample buffer, separated in SDS-PAGE and then subjected to Western blotting according to published procedure.

### Electron microscopy (EM)

Transmission Electron Microscopy (TEM) was essentially performed according to previously published protocol [41]. Following blocking step, the grids were incubated with the primary antibodies (anti-CagE, anti-CagA, anti-CagZ and anti-CagT at a dilution of 1:100) at 4°C in a humidified chamber for 2 hr followed by 1 hr incubation with colloidal gold conjugated Protein-A particles (15 nm, EY laboratories). The grids were negatively stained with 4% phospho-tungstate uranyl acetate (pH 4.0) and examined in a JEM-2100F (JEOL) transmission electron microscope.

### Scanning electron microscopy (SEM)

Wild-type *Hp* and mutant strains were grown on solid BHI agar plate as described in earlier section. AGS cells were co-cultured with *Hp* at MOI of 1:100 for 4 hr at 37°C in the presence of 5% CO_2_. Cells were fixed with 2.5% glutaraldehyde and dehydrated with graded ethanol. Dehydrated cells were chemically dried with HMDS (1,1,1,3,3,3,Hexamethyldirilazone), mounted onto sample stubs, grounded with silver paint at the sample edge and sputter-coated with palladium-gold before viewing with an Carl Zeiss Evo40 scanning electron microscope.

### Comparative sequence analysis, homology modelling and molecular dynamics simulation of CTD of CagE

Protein sequences of CagE and its nine homologues from different bacterial species, retrieved from UniProt database (http://www.uniprot.org/) were aligned by multiple sequence alignment program in Clustal Omega (http://www.ebi.ac.uk/Tools/msa/clustalo/). The alignment pattern obtained was analysed using Jalview (v2) to identify the conserved motifs and overall percentage identity of CagE with other homologues [48]. Out of nine homologues taken for sequence analysis, Crystal structure of CTD of only two proteins i.e. TrwB (*E*. *coli*, UniProt ID: Q04230) and tpsVirB4 (*T*. *Pseudethanolicus*, UniProt ID: B0KAW2) were available which had sequence identity of 16% and 14.63% respectively with CagE. To look more into the structural features of CagE, five multiple template based models of CTD (541 to 965, aa) of CagE were generated through homology modelling using Modeler (v-9.14) [49]. Template structures, TrwB (PDB ID: 1E9R) and tpsVirB4 (PDB ID: 4AG5) were obtained from Protein Data Bank (http://www.rcsb.org/). The model with lowest Discrete Optimized Protein Energy (DOPE) score was chosen and loops were refined. Eighteen residues from C-terminal were removed as those were forming coils with a free end. Further, to obtain stable conformation of the modelled structure, molecular dynamics simulation for 60 nano-seconds was performed using Gromacs (v-4.6.5) [50]. The simulation was performed using GROMOS96 force field in a cubical box where protein was solvated using SPC216 water molecules. Energy of the system using steepest descent method was minimised and this minimised structure represented the reference structure at time t = 0. After minimisation, the system was equilibrated under NVT (isothermal-isochoric) ensemble at 300K and NPT (isothermal-isobaric) ensemble to maintain 1 atm pressure. The time step was 2fs and co-ordinates were recorded every 10ps. Further analysis was performed using inbuilt Gromacs tools and graphs were generated using Grace (v-5.1.23) [http://plasma-gate.weizmann.ac.il/Grace/]. Structural validation was performed using ProSA-web and PDB sum [(https://www.ebi.ac.uk/thornton-srv/databases/pdbsum/Generate.html) [51]. Further structural comparison and visualization were performed using Pymol [52].

## Supporting Information

S1 Fig Schematic representation of the domain structure of CagE and purification profile. **(A)** Schematic representation of the domain structure of CagE. N, N terminus; C, C terminus, TM; putative trans-membrane domain, NBD, NTP binding domain, CagEN, residues 1 to 531; CagEC, residues 541 to 983. **(B)** SDS-PAGE showing the purified proteins after affinity purification. Molecular mass is indicated on the left side of the gel (kDa).
(DOCX)

S2 Fig ATPase activitiy of CagE N terminal domain. **(A)** Concentration-dependent ATPase activity of purified GST-CagEN (1–531). Lane C: reaction with GST only; lanes 1–3: reaction with increasing concentrations of protein as indicated. The positions of ATP and released Pi are indicated. **(B)** Time-dependent ATPase activity of purified GST-CagEN (1–531). Lane C: reaction with GST only; lanes 1–4: reaction with increasing time as indicated. **(C)** ATPase activity of purified wild-type and mutant proteins (Walker box A mutant) of GST-CagEC (541–983). Lane C reaction with GST alone, lanes 1–3 reaction with wild type protein with increasing concentrations, lanes 4–6 reaction with mutant protein with increasing concentrations. Positions of ATP and Pi are indicated. **(D)** Immune-depletion of ATPase activity of purified GST-CagEC (541–983). Lane 1 reaction with GST only, lanes 2–3 reaction with sample depleted with pre-immune IgG, lanes 4–5 reaction with sample depleted with immune IgG. **(E)** Immune-depletion of ATPase activity of purified GST-CagEN (1–531). Lane 1 reaction with GST only, lanes 2–3 reaction with sample depleted with immune IgG, lanes 4–5 reaction with sample depleted with pre-immune IgG.
(DOCX)

S3 Fig CagE is essentially required for the formation of surface pilus structure. **(A)** TEM and SEM images of wild-type *Hp* showing presence of surface pilus structure. **(B)** TEM and SEM images of *HpΔcagE* strain showing absence of surface pilus. **(C)** *HpΔcagX* strain showing absence of surface pilus structure in TEM and SEM images. Arrows indicate pilus structure in both SEM and TEM images respectively. *HpΔcagX* strain was used as a negative control for the absence of pilus. Scale bars indicate 100nm for TEM and SEM respectively.
(DOCX)

S4 Fig CagE is essential for CagA translocation into host gastric epithelial cells and IL-8 induction. **(A)** Western blots showing CagE dependent CagA phosphorylation in AGS cells infected with wild-type *Hp P12* and *cagE* complemented *P12ΔcagE/cagE*. Strains and antibodies used are indicated. CagE dependent CagA mediated humming bird phenotype is also being shown. Procedures followed are given in the materials and methods section. **(B)** CagE dependent IL-8 induction in AGS cells infected with wild-type *P12* and *cagE* complemented *P12ΔcagE/cagE* strains. IL-8 was measured by sandwich ELISA using *Hp* infected AGS cells culture supernatants as described in the materials and methods section. Strains used and % of IL-8 induction relative to wild-type strain is shown.
(DOCX)

S5 Fig Conserved motifs of Protein of VirB4 family shown in multiple sequence alignment. Conserved motifs: Motif A (red box, Walker Motif A), Motif B (pink box, Walker Motif B), Motif C (Black box) and Motif D (orange box) are shown. Conserved residues are highlighted in Blue with decreasing intensity as per residue identity across each column. Ten different VirB4 homologues were studied i.e. CagE_Hp: Uniprot ID Q48252; VirB4_Tps: Uniprot ID B0KAW2; VirB4_Rp: Uniprot ID Q9ZE45; VirB4_Rr: Uniprot ID Q7BLQ3; VirB4_Bh: Uniprot ID Q9R2W4; VirB4_Bm: Uniprot ID Q8YDZ4; lvhB4_Lp: Uniprot ID Q5X069; TraB_Ecol: Uniprot ID Q46698; TrwB_Ecol: Uniprot ID Q04230; Trwk_Ecol: Uniprot ID O50330.
(DOCX)

S6 Fig Molecular dynamics simulations results over 60 nano second time. **(A)** Graph showing RMSD of Protein with Starting structure. **(B)** Graph showing total Radius of gyration of protein around axis. **(C)** Number of Intra-protein Hydrogen bonds and **(D)** Residue wise RMSF for first 20 and last 40 nano second simulation period in Red and Black respectively.
(DOCX)

S7 Fig CagE is linked to outer membrane sub-complex proteins through CagV. **(A)** Western blots showing co-immunoprecipitation (Co-IP) of outer membrane core complex proteins CagT, CagM and CagX by anti-CagE and anti-CagV antibodies in *HpΔcagE* strain. **(B)** Western blots showing Co-IP of outer membrane core complex proteins CagT, CagM and CagX by anti-CagE and anti-CagV antibodies in *HpΔcagV* strain. Pre-immune serum was used as a negative control. The antibodies used in the western blots are indicated on the figure.
(DOCX)

S8 Fig Western blots showing specificity of the anti-CagE, anti-CagV and anti-Cagβ antibodies. **(A)** Specificity of anti-CagE mice antibody. **(B)** Specificity of anti-CagV rabbit antibody. **(C)** Specificity of anti-Cagβ mice antibody. Dilutions of antibodies used are shown on the figure. CagF was used as a loading control.
(DOCX)

S1 Table Sequence Identity of CagE of *Hp* with other homologues of VirB4. Overall Percent Identity is calculated by Clustal Omega. (DOCX)

S2 Table *Hp* and *E*. *coli* strains used in the present study. (DOCX)

S3 Table List of sets of primers pair used for amplification of gene fragment for construction of recombinant plasmids. (DOCX)

S4 Table Oligonucleotides used in the present study. Alphabets ‘f’ and ‘r’ indicate forward and reverse primers used to construct plasmids.
(DOCX)

## Abbreviations

RMSD: Root mean square deviation
RMSF: Root mean square fluctuation
H-BOND: Hydrogen bond
ns: nano second
ps: pico second
fs: femto second
MD: Molecular Dynamics
