# Supplementary material for: Biochemical Analysis of CagE: A VirB4 Homologue of Helicobacter pylori Cag-T4SS
Source: PLoS One. 2015 Nov 13;10(11):e0142606. doi: 10.1371/journal.pone.0142606 (PMC4643968; doi:10.1371/journal.pone.0142606)
Supplement: S1 Table — (DOCX) [file pone.0142606.s009.docx]

| SL No. | Uniprot ID (Protein name) | Organism | Identity (%) |
| --- | --- | --- | --- |
| 01 | B0KAW2(VirB4 components-like) | *Thermoanaerobacter pseudethanolicus* | 14.63 |
| 02 | Q7BLQ3 (VirB4) | *Rhizobium radiobacter* | 22.66 |
| 03 | Q8YDZ4 (VirB4) | *Brucella melitensis* | 26.65 |
| 04 | Q9R2W4 (VirB4) | *Bartonella henselae* | 25.85 |
| 05 | Q9ZE45 (VirB4) | *Rickettsia prowazekii* | 24.52 |
| 06 | Q5X069 (Vir homologue) | *Legionella pneumophila* | 21.78 |
| 07 | Q04230 (TrwB) | *Escherichia coli* | 16.00 |
| 08 | O50330 (TrwK) | *Escherichia coli* | 24.81 |
| 09 | Q46698 (TraB) | *Escherichia coli* | 21.34 |
