# Supplementary material for: Biochemical Analysis of CagE: A VirB4 Homologue of Helicobacter pylori Cag-T4SS
Source: PLoS One. 2015 Nov 13;10(11):e0142606. doi: 10.1371/journal.pone.0142606 (PMC4643968; doi:10.1371/journal.pone.0142606)
Supplement: S2 Table — (DOCX) [file pone.0142606.s010.docx]

| **Strains** | **Descriptions** | **References** |
| --- | --- | --- |
| *Hp26695* | *Helicobacter pylori* wild type strain | Tomb et. al.1997 |
| *P12* | *Helicobacter pylori* wild type strain | Fischer et al, 2010 |
| *P12ΔcagE* | Deletion mutant for *hp0544* (*cagE*) | This study |
| *P12ΔcagE/cagE* | Complemented strain of wild-type *cagE* gene | This study |
| *Hp26695∆cagE* | Deletion mutant for *hp0544* (*cagE*) | This study |
| *Hp26695∆cagZ* | Deletion mutant for *hp0526* (*cagZ*) | Kumar et al. 2013 |
| *Hp26695∆cagV* | Deletion mutant for *hp0530* (*cagV*) | Kumar et al. 2013 |
| *Hp26695∆cagT* | Deletion mutant for *hp0532* (*cagT*) | Fischer et. al. 2001 |
| *Hp*26695∆*cagX* | Deletion mutant for *hp0528* (*cagX*) | Kumar et al. 2013 |
| *Hp26695∆cagI* | Deletion mutant for *hp0540* (*cagI*) | Kumar et al. 2013 |
| *Hp* 26695∆*cagH* | Deletion mutant for *hp0541* (*cagH*) | Kumar et al. 2013 |
| *Hp26695∆cagM* | Deletion mutant for *hp0537* (*cagM*) | Kumar et al. 2013 |
| *Hp26695∆cagY* | Deletion mutant for *hp0527* (*cagY*) | Kumar et al. 2013 |
| *Hp26695∆cagδ* | Deletion mutant for *hp0522* (*cagδ*) | Kumar et al. 2013 |
| *E. coli BL21(DE3)* | Host for co-expression | Novagen |
| *E. coli DH5α* | General cloning host | Life technologies |
