# Supplementary material for: Biochemical Analysis of CagE: A VirB4 Homologue of Helicobacter pylori Cag-T4SS
Source: PLoS One. 2015 Nov 13;10(11):e0142606. doi: 10.1371/journal.pone.0142606 (PMC4643968; doi:10.1371/journal.pone.0142606)
Supplement: S3 Table — (DOCX) [file pone.0142606.s011.docx]

| **Construct name** | **Primer pair used for amplification** | **Cloning site** |
| --- | --- | --- |
| pGEX-∆N140*cagE* | F*cagE*B/R*cagE*S | BamHI/SalI |
| pACYC-*cagV*A | F*cagV*B/R*cagV*H | BamHI/HindIII |
| pGEX-*cagE*∆C452 | F*cagE*B/R*cagE*S | BamHI/SalI |
| pET-*cagβ*ΔN170 | FΔ170N*cagβ*N/R*cagβ*B | NdeI/BamHI |
| pGEX-∆N540*cagE* | F*cagE*B/R*cagE*S | BamHI/SalI |
| pET-*cagV* | F*cagV*N/R*cagV*H | NcoI/HindIII |
| mutCagE | fmutCagE/rmutCagE |  |
| pBS-*cag*8A  pBS-*cag8A*∆*cagE*/*CatGc* | f*cag*8AN/r*ag*8AK  F*cag8A∆cagE*B/R*cag8A∆cagE*X | KpnI/NotI  BamHI/XhoI |
| pJP99CagAP | F*cagA*PS/R*cagA*PB | SalI/BamHI |
| pJP99CagAP-CagE | F*cagA*P-cagEB/R*cagA*P-cagEK | BamHI/KpnI |
