# Supplementary material for: Biochemical Analysis of CagE: A VirB4 Homologue of Helicobacter pylori Cag-T4SS
Source: PLoS One. 2015 Nov 13;10(11):e0142606. doi: 10.1371/journal.pone.0142606 (PMC4643968; doi:10.1371/journal.pone.0142606)
Supplement: S4 Table — Alphabets ‘f’ and ‘r’ indicate forward and reverse primers used to construct plasmids. (DOCX) [file pone.0142606.s012.docx]

| fΔ140N*cagE* BamHI | 5’-CCGGATCCATGGCTAATGACATCCACTTA-3’ |
| --- | --- |
| r*cagE* SalI | 5’-ATGCGTCGACTTAATACTCCTTTATTTGTTG-3’ |
| f*cagV* BamHI | 5’-CGGGATCCATGTTAGGGAAAAAAAACG-3’ |
| r*cagV* HindIII | 5’-CCCAAGCTTCTATTTATTTAATGCCTTATTTTTTG-3’ |
| fmutCagE | 5’- TGGCTCAACCGGTTCAGGTGCGACAGTGTTTATGTCAATGA-3’ |
| rmutCagE | 5’- TCATTGACATAAACACTGTCGCACCTGAACCGGTTGAGCCA-3’ |
| f*cagE* BamHI | 5’- CCGGATCCGTGTTTGTGGCAAGCAAACAA-3’ |
| r*cagE∆452C* SalI | 5’- ATGCGTCGACTTAAGCAAAATTATTAGAAGTTACATCAAA-3’ |
| fpJP99*CagAP*SalI | 5’-ATGCGTCGACACTATGGTAAGTATGACA-3’ |
| rpJP99*CagAP*BamHI | 5’-CCGGATCCTGTTTCTCCTTACTATACCTA-3’ |
| fpJP99*CagAP- CagE*BamHI | 5’-CCGGATCCGTGTTTGTGGCAAGCAAAC-3’ |
| rpJP99*CagAP- CagE*KpnI | 5’-GCCCGGTACCTTAATACTTCCTTTATTTGTTGATAC-3’ |
| fΔ540N*cagE* BamHI | 5’-CCGGATCCGCTGATTTCATCGCTATG-3’ |
| fΔ170N*cagβ*NdeI | 5’-GGCCCCATATGCGGACTAGAGATATAGGAGCG-3’ |
| r*cagβ*BamHI | 5’-CCGGATCCTCACAGTTCACTTGAACCCACAGG-3’ |
| f*cagV*BamHI | 5’-CGGGATCCATGTTAGGGAAAAAAAAACG-3’ |
| rcag*V*HindIII | 5’-CCCAAGCTTCTATTTATTTAATGCCTTATTTTTTG-3’ |
| f*cag8*ANotI | 5’-GGTGGCGGCCGCATCTGAGAATCCTATACG-3’ |
| r*cag8*AKpnI | 5’- GCCCGGTACCATGCAAGGTGAGTGCATG-3’ |
| f*cagE* BamHI | 5’-GGGGATCCGTCGACCAAATAAAGGAGTATTAAA-3’ |
| r*cagE* XhoI | 5’-ACCGCTCGAGGCTTGCCACAAACACCCCT-3’ |
| f*cat* XhoI | 5’-CCGCTCGAGTTGTTGATGGGGCAGGCA-3’ |
| r*cat* BamHI | 5’-GCGGATCCTTATTTATCTCTGACAAGAG-3’ |
