# Supplementary material for: Biochemical Analysis of CagE: A VirB4 Homologue of Helicobacter pylori Cag-T4SS
Source: PLoS One. 2015 Nov 13;10(11):e0142606. doi: 10.1371/journal.pone.0142606 (PMC4643968; doi:10.1371/journal.pone.0142606)
Supplement: Supplementary file 5 [file pone.0142606.s001.docx]

A

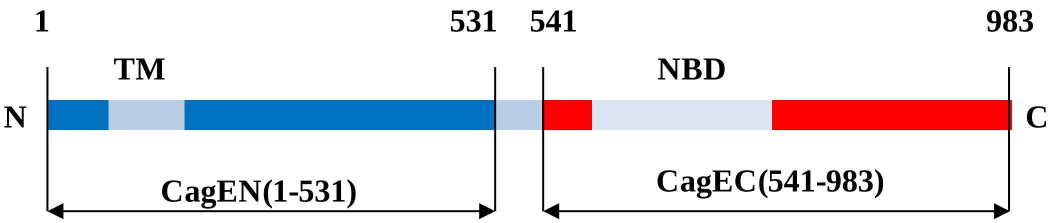
1
531
541
983
TM
NBD
N
C
CagEN(1-531)
CagEC(541-983)

B

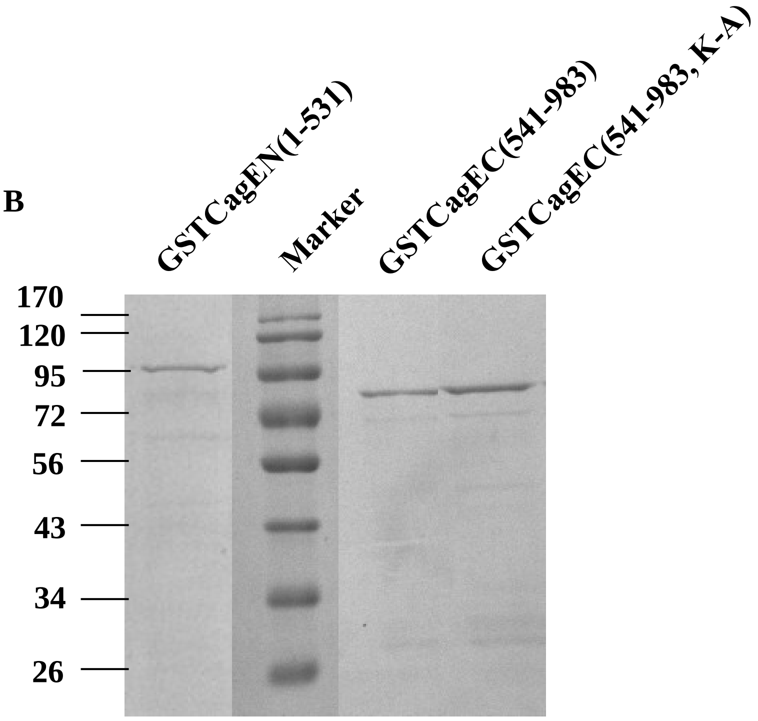
GSTCagEN(1-531)
Marker
GSTCagEC(541-983)
GSTCagEC(541-983, K-A)
170
120
95
72
56
43
34
26
