# Supplementary material for: Biochemical Analysis of CagE: A VirB4 Homologue of Helicobacter pylori Cag-T4SS
Source: PLoS One. 2015 Nov 13;10(11):e0142606. doi: 10.1371/journal.pone.0142606 (PMC4643968; doi:10.1371/journal.pone.0142606)

TEM **SEM**

A

26695

B

26695ΔcagE

C

26695ΔcagX

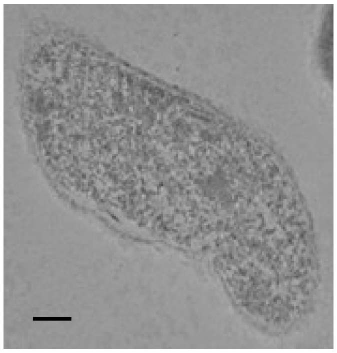

Supplement: Supplementary file 7 [file pone.0142606.s003.docx]
