# Supplementary material for: Biochemical Analysis of CagE: A VirB4 Homologue of Helicobacter pylori Cag-T4SS
Source: PLoS One. 2015 Nov 13;10(11):e0142606. doi: 10.1371/journal.pone.0142606 (PMC4643968; doi:10.1371/journal.pone.0142606)
Supplement: Supplementary file 8 [file pone.0142606.s004.docx]

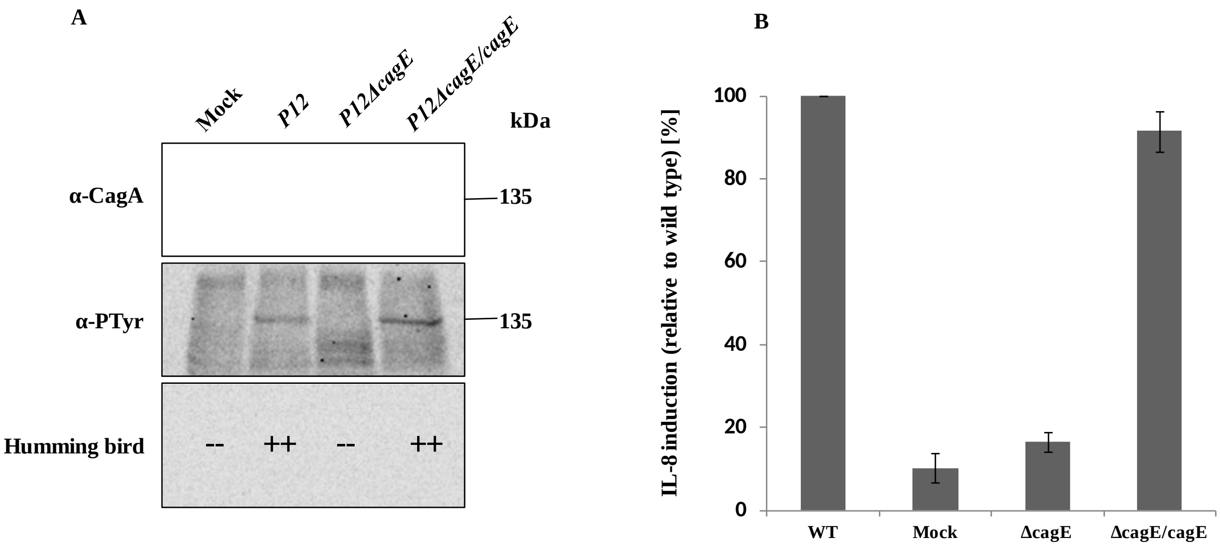
A
Mock
P12
P12ΔcagE
P12ΔcagE/cagE
kDa
α-CagA
135
α-PTyr
135
Humming bird
--
++
--
++
B
IL-8 induction (relative to wild type) [%]
100
80
60
40
20
0
WT
Mock
ΔcagE
ΔcagE/cagE
