# Supplementary material for: Biochemical Analysis of CagE: A VirB4 Homologue of Helicobacter pylori Cag-T4SS
Source: PLoS One. 2015 Nov 13;10(11):e0142606. doi: 10.1371/journal.pone.0142606 (PMC4643968; doi:10.1371/journal.pone.0142606)
Supplement: Supplementary file 10 [file pone.0142606.s006.docx]

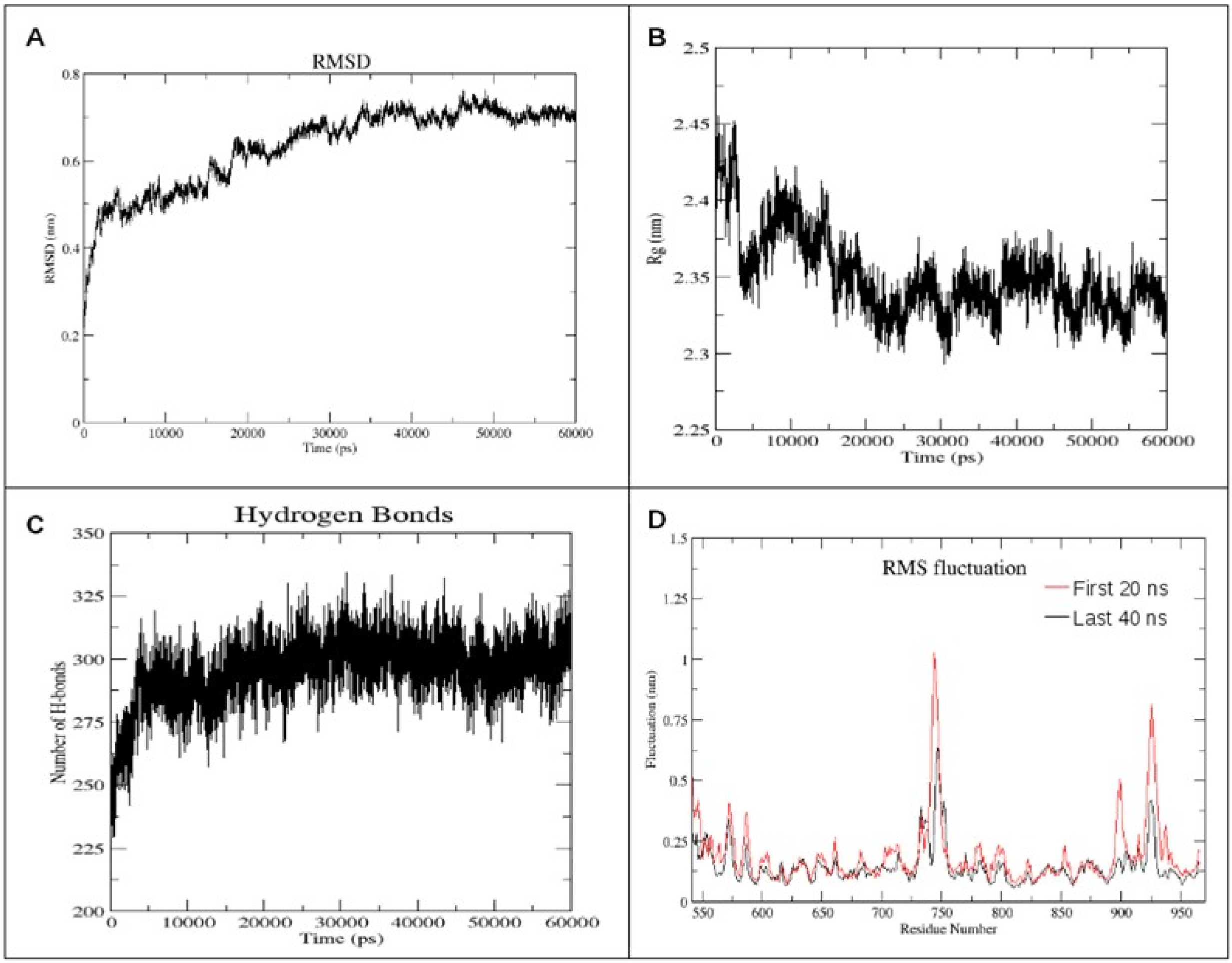

A
RMSD
RMSD (nm)
Time (ps)
B
Rg (nm)
Time (ps)
C
Hydrogen Bonds
Number of H-bonds
Time (ps)
D
RMS fluctuation
First 20 ns
Last 40 ns
Fluctuation (nm)
Residue Number
