# Supplementary material for: Biochemical Analysis of CagE: A VirB4 Homologue of Helicobacter pylori Cag-T4SS
Source: PLoS One. 2015 Nov 13;10(11):e0142606. doi: 10.1371/journal.pone.0142606 (PMC4643968; doi:10.1371/journal.pone.0142606)
Supplement: Supplementary file 11 [file pone.0142606.s007.docx]

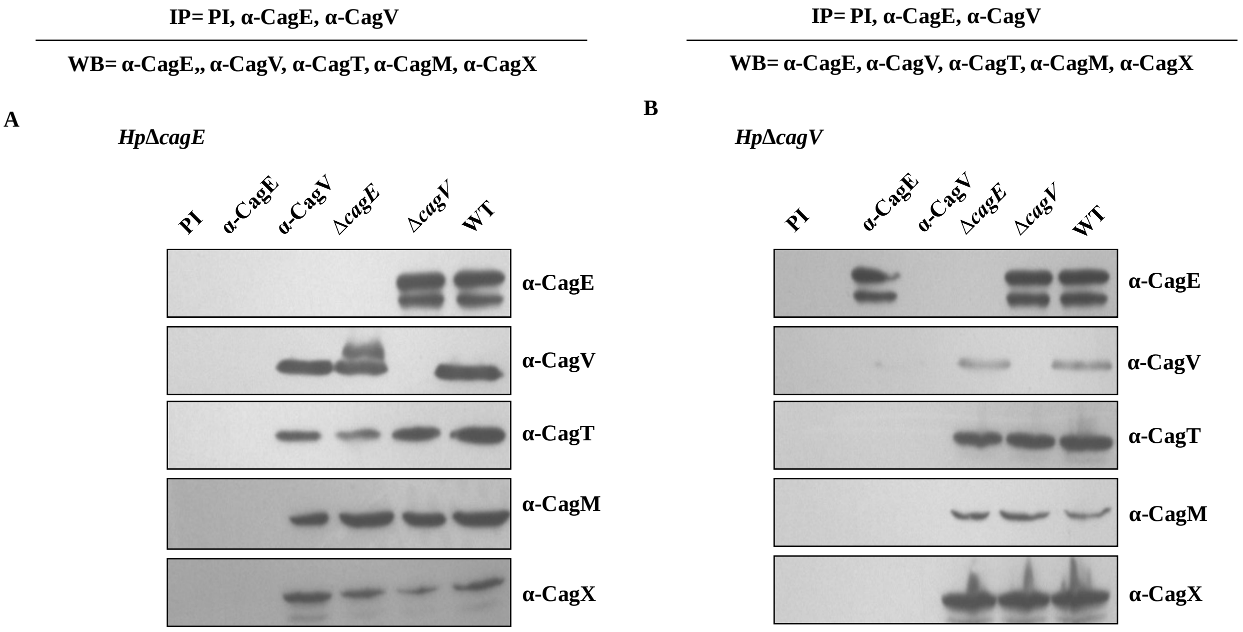
IP= PI, α-CagE, α-CagV
WB= α-CagE,, α-CagV, α-CagT, α-CagM, α-CagX
A
HpΔcagE
PI
α-CagE
α-CagV
ΔcagE
ΔcagV
WT
α-CagE
α-CagV
α-CagT
α-CagM
α-CagX
IP= PI, α-CagE, α-CagV
WB= α-CagE, α-CagV, α-CagT, α-CagM, α-CagX
B
HpΔcagV
PI
α-CagE
α-CagV
ΔcagE
ΔcagV
WT
α-CagE
α-CagV
α-CagT
α-CagM
α-CagX
