# Supplementary material for: Biochemical Analysis of CagE: A VirB4 Homologue of Helicobacter pylori Cag-T4SS
Source: PLoS One. 2015 Nov 13;10(11):e0142606. doi: 10.1371/journal.pone.0142606 (PMC4643968; doi:10.1371/journal.pone.0142606)
Supplement: Supplementary file 12 [file pone.0142606.s008.docx]

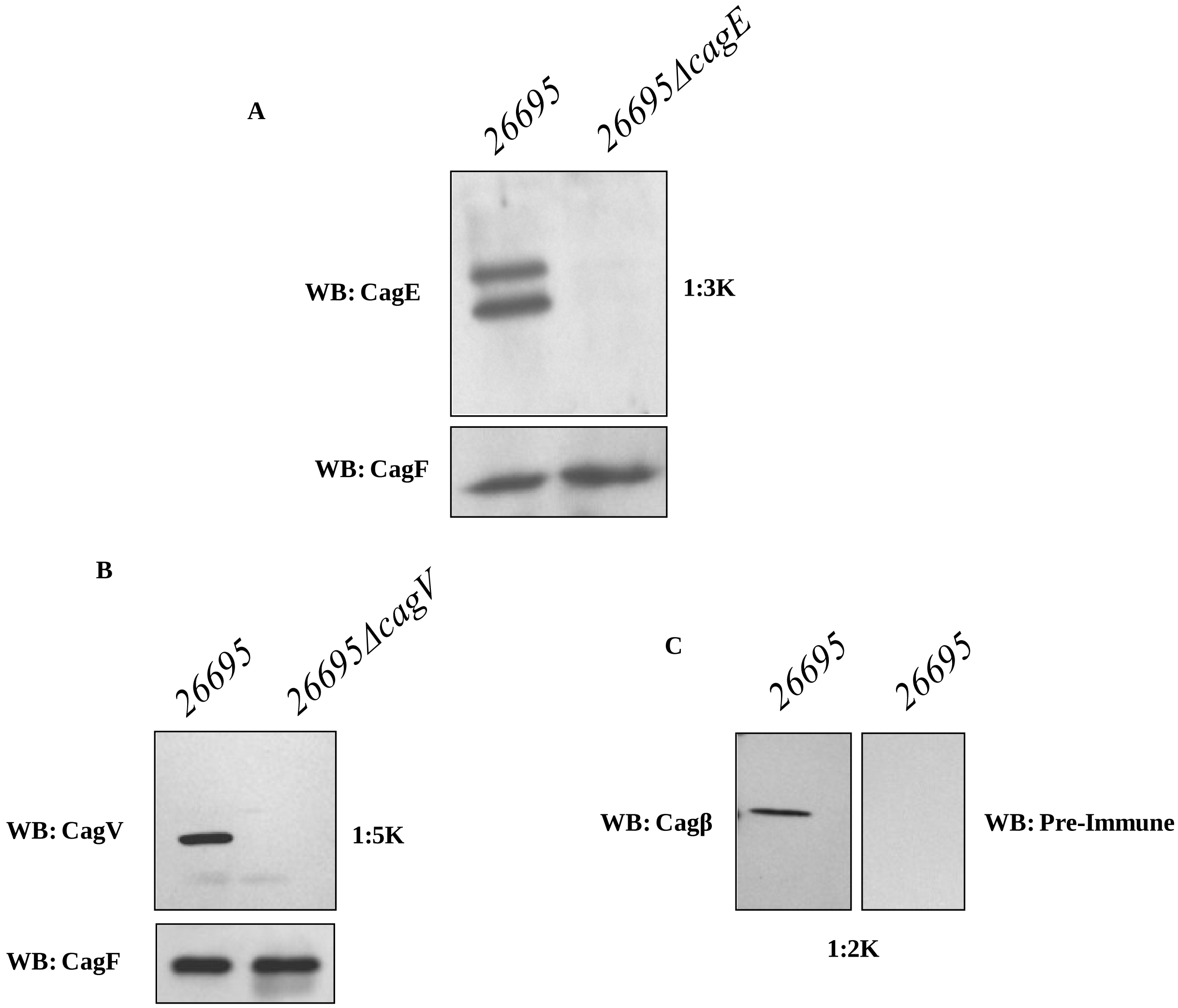
A
26695
26695ΔcagE
WB: CagE
1:3K
WB: CagF
B
26695
26695ΔcagV
WB: CagV
1:5K
WB: CagF
C
26695
26695
WB: Cagβ
WB: Pre-Immune
1:2K
